# Supplementary material for: Migration of co-cultured endothelial and smooth muscle cells on material surfaces
Source: Regen Biomater. 2026 May 15;13:rbag087. doi: 10.1093/rb/rbag087 (PMC13229579; doi:10.1093/rb/rbag087)
Supplement: rbag087_Supplementary_Data [file rbag087_supplementary_data.zip › Manuscript_RB_Revised_Marked Ver5.pdf]

Migration of co-cultured endothelial and smooth muscle cells on material surfaces

*Runjia Shen, Yanshuang Zhang, Ziyue Zhang, Qiong Liu, Jiandong Ding**

State Key Laboratory of Molecular Engineering of Polymers, Department of Macromolecular Science, Fudan University, Shanghai 200438, China

* Corresponding author. Email: jdding1@fudan.edu.cn (JD Ding)

Abstract

Surface endothelialization of a medical device after implantation into the cardiovascular system depends upon competitive migration between endothelial cells (ECs) and smooth muscle cells (SMCs). While both cell types have been extensively studied, the relative migration under mixed co-culture condition remains elusive. Herein, we investigate the migration of ECs and SMCs on tissue culture plates under different culture modes. Compared with mono-cultures, mixed co-culture slows down the migration of both cell types. Our analyses suggest that this reduced migration likely involves intensified intercellular interactions and expanded cell coverage during the mixing of two entities with different sizes. We find that the mixed co-culture may alter the relative migration between ECs and SMCs. We further examine the relative migration of the two cell types on arginine-glycine-aspartate (RGD) nanoarrays prepared using block copolymer micelle nanolithography, and show that nanopatterns with varied RGD nanospacings (29-121 nm) can significantly tune the relative migration rate, effectively reversing the migratory dominance of one cell type over the other. This study sheds new insights on the behavior of co-cultured cells on biomaterials with modified surfaces.

Keywords: Biomaterials, Cell migration, Co-culture, RGD nanopattern, Endothelial cell, Smooth muscle cell, Cell adhesion, Surface patterning

Graphic Abstract

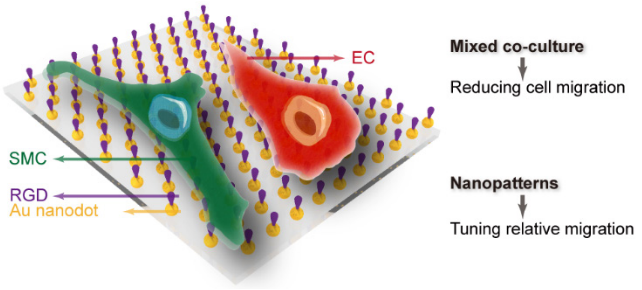

Introduction

The vascular tissue is primarily composed of two cell types, endothelial cells (ECs) and smooth muscle cells (SMCs), whose interactions are essential for vascular development, homeostasis, and regeneration, as well as for pathological processes such as atherosclerosis and restenosis [1, 2]. Their relative migration is critical for vascular repair and remodeling, as it directly determines neointimal formation [3, 4]. Therefore, regulating EC and SMC migration is imperative for the design of vascular grafts and cardiovascular stents that aim to promote rapid endothelialization while suppressing excessive SMC infiltration [5-8]. Achieving this goal requires not only modulating the migration of individual cell types but also understanding how interactions between the two cell types regulate their relative migration.

It is well established that biomaterials can profoundly influence cellular behaviors [9-13]. Considerable efforts have been devoted to understanding how material cues [14-16], including substrate stiffness [17-20], surface topography [21-24], chemical composition [25, 26], ligand density and distribution [27, 28] regulate cell migration. Most prior research has focused primarily on monocultures of a single cell type, providing only a limited view of the complexity in the case of co-existing vascular cell types [29, 30]. Growing evidence suggests that interactions between ECs and SMCs play a crucial role in regulating cellular functions [31]. For instance, factors released by EC can inhibit SMC migration [32], while cytokines secreted by SMCs regulate endothelial barrier integrity and angiogenic behavior. Despite these advancements, the relative migration of ECs and SMCs under mixed co-culture condition remains poorly understood, limiting the rational design of biomaterial surfaces for directing multicellular migration in vascular regeneration. Nanoscale engineering of biomaterial surfaces has emerged as an effective strategy to regulate cell behaviors [33, 34]. For instance, the spatial arrangement of ligands at the nanoscale strongly influences integrin clustering, focal adhesion maturation, and cytoskeletal organization [35]. Despite this, how nanoscale ligand spacing affects the relative migration of multiple cell types in co-culture remains largely unexplored.

Here, we investigate the migratory behaviors of ECs and SMCs under mono-culture and mixed co-culture conditions, as schematically illustrated in Figure 1, and explore the physical and biochemical mechanisms governing their relative migration. In order to visualize living cells under a fluorescence microscope, ECs and SMCs were transduced with lentiviral vectors encoding LifeAct-tagRFP and LifeAct-EGFP, respectively, enabling fluorescent labeling of F-actin. The mixed co-culture was found to significantly reduce the migration of both cell types, particularly ECs. Our results suggest that this phenomenon likely stems from putative enhanced paracrine signaling (a biological effect) and enlarged cell coverage arising from close packaging of entities of different sizes (a geometry effect). Furthermore, by employing arginine-glycine-aspartate (RGD) nanoarrays with tunable nanospacings, we show that the nanoscale ligand organization can effectively regulate the relative migration rate of ECs and SMCs and even effectively reverse the migratory dominance of one cell type over the other. Together, these results deepen our understanding of how vascular cells migrate on biomaterial surfaces and highlight ligand nanospacing as a key parameter for tuning multicellular behaviors.

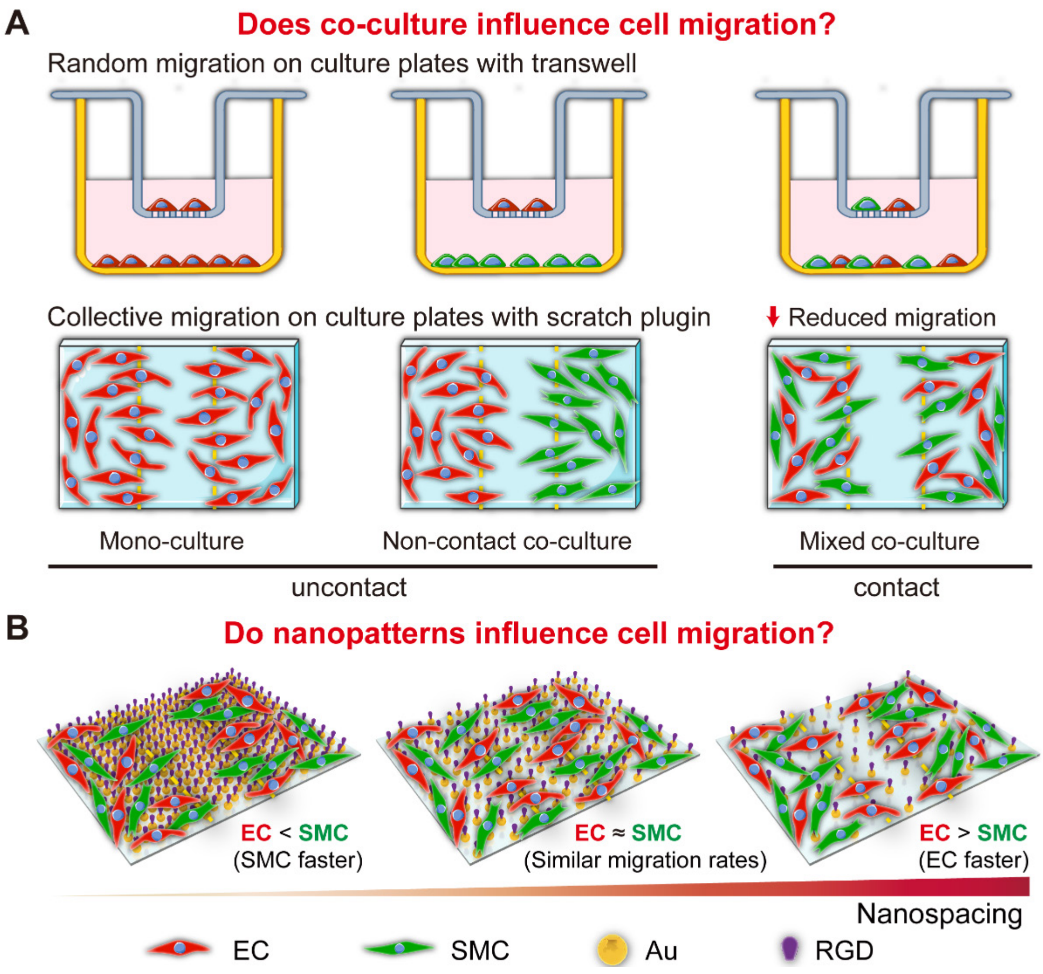

Figure 1. Schematic illustration of the experimental design investigating the effects of co-culture and nanopatterns on migration of endothelial cells (ECs) and smooth muscle cells (SMCs). (**A**) Random migration assays for comparison among mono-culture, non-contact co-culture, and mixed co-culture conditions. Mono-culture contains only ECs (red); non-contact co-culture allows paracrine interaction between ECs and SMCs without physical contact; mixed co-culture permits both paracrine and physical cell–cell interactions. The total cell density was kept constant across groups. Collective migration configurations on culture plates under the same three culture conditions. The schematics illustrate how ECs and SMCs exhibit distinct collective migration behaviors depending on whether intercellular communication is absent, paracrine only, or direct-contact mediated. (**B**) Schematic representation of cell migration on nanopatterned surfaces with different RGD ligand nanospacings. The model illustrates the possibility that ECs and SMCs respond to nanoscale spatial cues and intercellular interactions on Au nanopatterns, highlighting the potential effects of nanopatterning and co-culture on relative cell migration.

Materials and methods

Construction of LifeAct-RFP-labeled ECs and LifeAct-GFP-labeled SMCs by lentiviral transduction

Two cell lines were examined, human umbilical vein endothelial cells (HUVECs, SCSP-5285), obtained from the Chinese Academy of Sciences, and human aortic smooth muscle cells (HASMCs, HUM-iCell-c010), obtained from iCell Bioscience Inc. (Shanghai, China). Both cell types were maintained at 37 °C in a humidified incubator (Binder, Germany) with 5% CO_2. HUVECs were cultured in high-glucose Dulbecco's modified Eagle medium (DMEM) supplemented with 10% fetal bovine serum (FBS, Sciencell) and 1% penicillin–streptomycin. HASMCs were cultured in complete medium (iCell Bioscience Inc., Shanghai) according to the manufacturer's instructions. For all experiments, a 0.5:0.5 mixture of the two media was used as the control.

To enable real-time visualization by live-cell workstation and subsequent quantification of cell migration, ECs and SMCs were genetically labeled with fluorescent markers. Lentiviral vectors encoding LifeAct-RFP (Cat. No. H5674) and LifeAct-GFP (Cat. No. H6189) were purchased from OBiO Technology (Shanghai, China) and used to label F-actin in ECs and SMCs, respectively. Cells were transduced following standard lentiviral transduction for 12 h in the presence of 10 μg/mL polybrene (OBiO Technology, Shanghai, China). Transduction efficiency was assessed by flow cytometry. These labeled cell lines were subsequently used for migration experiments.

Collective migration assay of ECs and SMCs

To investigate the collective migration behaviors of ECs and SMCs under different culture conditions, a scratch assay was performed using Ibidi culture insert (Cat. No. 80209) to create an initial gap of 500 μm. The sterilized insert was first adhered to substrate surface. For mono-culture conditions, 70 μL of EC or SMC suspension was added to both compartments of the insert at a density of 3×10^5 cells per milliliter. For non-contact co-culture condition, ECs and SMCs were seeded separately into the two sides of the insert at the same density. For mixed co-culture condition, ECs and SMCs were premixed at a ratio of 0.5:0.5 (v/v) prior to seeding, and 70 μL of the mixed cell suspension was added to each compartment. The total seeding density was kept consistent across all groups to ensure comparability.

Cells were allowed to attach and spread on substrate surfaces for 8 h in a humidified incubator. The insert was then removed gently using tweezers. Cell nuclei were subsequently stained with Hoechst 33342 (Beyotime Biotechnology, Cat. No. C1025) for 5 min, followed by three washes with phosphate-buffered saline (PBS, 3 × 10 min). Two milliliters of 0.5:0.5 mixed culture medium, as described above, were added to each group. Cell migration was observed for 12 h using a live-cell imaging workstation, whose temperature and atmospheric conditions were set to be the same as a cell incubator. Time-lapse images were collected for quantitative analysis with ImageJ (National Institutes of Health, USA; https://imagej.net/).

Random migration assay of ECs and SMCs

To investigate the random migration of two cell types under different culture conditions, a transwell insert system was used to establish the non-contact co-culture configuration, as schematically illustrated in Figure 1A. Here, cell migration was quantified by time-lapse imaging of cells migrating on the substrate rather than by transmembrane migration across the insert. For consistency, the same insert setup was used for all groups. Cell suspensions were added at a total density consistent across groups, with 2

mL suspension added to the lower chamber and 0.5 mL to the upper chamber. Cells were seeded according to the specific culture condition for each group, and all groups were cultured in a 0.5:0.5 mixed medium. After incubation on the corresponding surfaces for 8 h, cell nuclei were stained with Hoechst 33342 for following analysis.

Random migration of ECs and SMCs was monitored using a living-cell workstation, with images captured at 30 min intervals, lasting for 12 h. The random migration rate of ECs and SMCs was evaluated by time-lapse live-cell imaging and analyzed using the ImageJ plugin TrackMate-Manual Tracking. The nucleus center of each cell was manually tracked at each time point, enabling the extraction of individual cell positions and the determination of the migration trajectory. $\boldsymbol{l}_i$ is defined as the displacement between consecutive time points t_{i-1} and t_i. Migration speed, V_{contour}, is calculated from the contour length over time. The end-to-end displacement vector is written as $\boldsymbol{R}(t_n) - \boldsymbol{R}(t_0)$.

CCK-8 cell viability assay

Cell metabolic activity was quantified using a cell counting kit-8 (CCK-8, Beyotime) assay. For experimental consistency, all groups were uniformly established using a transwell system. Cells were seeded at an initial density of 2×10^5 cells per milliliter into both the upper inserts and lower wells according to the experimental design, maintaining identical medium composition and cell densities over all groups. Five groups were established: EC/EC (endothelial cells in both upper and lower chambers), SMC/EC (smooth muscle cells in the upper chamber and endothelial cells in the lower chamber), SMC/SMC, EC/SMC, and Mixed/Mixed (a 0.5:0.5 mixture of ECs and SMCs in both compartments). After 12 h of culture, the medium was replaced with fresh medium containing 10% (v/v) CCK-8 reagent and incubated at 37 °C in the dark for 1 h. Blank wells without cells were included to correct background absorbance. The absorbance or optical density was measured at 450 nm (OD_{450}) using a microplate reader. After background subtraction, OD_{450} values were used to represent the cellular metabolic activity of each group.

Fabrication of non-nano surfaces with densely grafted RGD ligands

Non-nano surfaces were prepared by sequential sputtering of chromium (Cr, 60 s × 3 cycles) and gold (Au, 60 s × 3 cycles) onto clean and dry substrates using a sputter coater (SBC12, KYKY, China) under vacuum conditions. Prior to cell seeding, the non-nano surfaces were functionalized with the cell-adhesive RGD peptide c(RGDfK)–SH (Shanghai Jetide Biotech Co., Ltd., Cat. No. JT-96868) through the thiol end group of c(RGDfK)−SH at 4°C for 8 h. After the grafting process, the surfaces were thoroughly rinsed with PBS to remove unbound peptides, ensuring they were ready for subsequent experiments.

Fabrication of RGD nanoarrays on a nonfouling poly(ethylene glycol) (PEG) background

Au nanoarrays were fabricated on substrates by block copolymer micellar nanolithography using polystyrene-*block*-poly(2-vinylpyridine) (PS-*b*-P2VP, Polymer Source) as the template polymer, following the published protocol [36, 37]. Due to the amphiphilicity of the block copolymer, micelles with a core–corona structure formed after dissolving copolymer in toluene (Sigma) under constant stirring for 24 h. After that, hydrogen tetrachloroaurate (III) hydrate ($HAuCl_4 \cdot 3H_2O$, Alfa Aesar) was added to the solution as a gold precursor, and the solution was stirred for an additional 24 h.

Substrates were first cleaned by wipes and pretreated with piranha solution (98% H_2SO_4:30% H_2O_2 = 3:1, v/v) for 2 hours. Afterwards, they were rinsed thoroughly with Milli-Q water, sonicated for 10 min three times, and dried under nitrogen. The clean

substrates were then dipped in the micelle solution to form a monolayer of micelles on the surface. After complete evaporation of toluene under dry conditions, the substrate surfaces were treated with oxygen plasma (DT-03s, OPS Plasma, China) at 150 W for 1 h to remove the polymer template and an Au nanoarray was obtained.

To create a nonfouling background, the Au nanoarrays were then functionalized with M-PEG-Si(OMe)$_3$ (Gelest). M-PEG-Si(OMe)$_3$ was dissolved in xylene at a final concentration of 0.28 mg/mL, and triethylamine (1 mL per 100 mL of solution) was added as a catalyst. The Au nanoarrays were immersed in this solution at 65°C for at least 24 h. After PEGylation, the substrates were rinsed thoroughly with xylene and then ethanol to remove unbound molecules, followed by drying under nitrogen.

Then, the RGD peptides were grafted onto the Au nanodots by immersing the nanopattern in an aqueous solution of c(RGDfK)−SH at 4 °C overnight. After RGD grafting, the surfaces were rinsed thoroughly with PBS to remove unbound peptide and used immediately for subsequent experiments. This procedure resulted in RGD-functionalized Au nanoarrays on a nonfouling PEG background, providing a well-defined nanopattern with high cell-adhesion contrast.

Surface characterization of nanopatterns

The morphology of the as-prepared RGD nanopatterns was examined using a field-emission scanning electron microscope (FESEM, Gemini 560, Zeiss) at an accelerating voltage of 1.5 kV. The average nanospacing of the Au nanodots was calculated from the FESEM images using ImageJ software. The average nanospacing was calculated from three representative areas on each of three independent samples.

Observation of cell behaviors on RGD nanopatterns

To evaluate the effect of ligand nanospacing on cell viability, cells were seeded on nanopatterned substrates or non-nanopatterned control surfaces (Non-nano) at an initial density of 2×10^5 cells per milliliter and cultured under standard conditions. After 12 h of culture, CCK-8 working solution was added directly to each well (10% v/v), followed by incubation at 37 °C for 1 h protected from light. Absorbance at 450 nm was recorded, and blank wells were used for background correction. For quantitative comparison across substrates, the background-corrected OD_{450} of each nanopatterned group was normalized to that of the Non-nano control, which was defined as 1. The resulting values were reported as normalized average cell viability. All measurements were performed with at least four independent replicates.

For experiments of cells on nanopatterned surfaces, RGD nanopatterns and non-nano control surfaces were immobilized on standard culture plates. All substrates were sterilized with 75% ethanol (30 min × 2) and thoroughly rinsed with PBS (30 min × 2) to remove residual ethanol before cell seeding. Then, both cell types were seeded according to the experiment design, keeping consistent cell density for all groups.

Statistical analysis

All image analyses were performed using ImageJ software. In the bar graphs, individual data points represent measurements from single cells. Statistical significance was determined by one-way ANOVA. The p value less than 0.05 was taken as the criterion of a significant difference.

Results

Construction of fluorescent living cells

To distinguish the two cell types during live-cell imaging and enable subsequent quantitative analysis of cell migration, ECs and SMCs were transduced with lentiviral vectors encoding LifeAct-RFP and LifeAct-GFP, respectively, as illustrated in Figure 2A. According to flow cytometric analysis, 98.4% of ECs ($n > 5000$) were RFP-positive and 98.8% of SMCs ($n > 5000$) were GFP-positive, as shown in Figure 2B, indicating that most ECs and SMCs were successfully labeled. Fluorescence microscopy revealed distinct optical signals in ECs and SMCs, with actin-associated fluorescence observed under mono-culture and mixed co-culture conditions, enabling clear identification of each cell type during subsequent analyses (Figure 2C). In addition, bright-field imaging and short tandem repeat (STR) profiling confirmed that lentiviral transduction did not alter cell morphology or genetic identity, as evidenced in Figures S1 and S2.

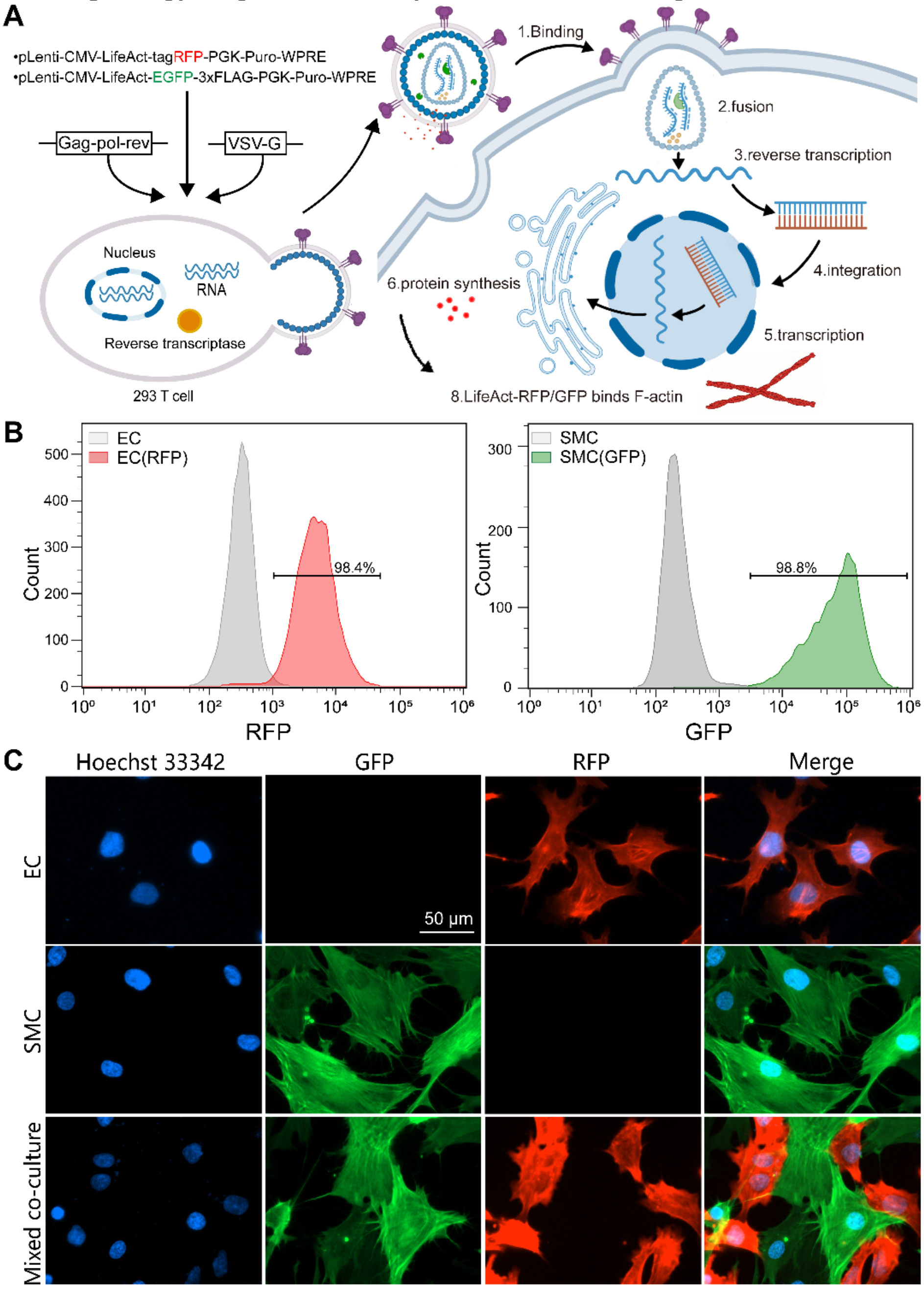

Figure 2. Gene-engineering construction of fluorescent living cells to enable real-time visualization and thus quantifying cell migration. (**A**) Schematic presentation of the lentiviral transduction procedure used to introduce LifeAct-tagRFP and LifeAct-EGFP

constructs for F-actin labeling in ECs and SMCs. (**B**) Flow cytometry analysis validating high transduction efficiency, with 98.4% of ECs ($n > 5000$) positive for RFP and 98.8% of SMCs ($n > 5000$) positive for GFP. Together, these results confirm the reliable establishment of dual-color labeling for distinguishing ECs and SMCs in subsequent co-culture experiments. (**C**) Representative fluorescence micrographs of the lentivirus-transduced cells showing nuclei (Hoechst 33342, blue), LifeAct-RFP expressing endothelial cells (ECs, red), LifeAct-GFP expressing smooth muscle cells (SMCs, green). The merged images demonstrate specific and stable expression of the fluorescent labels in their respective cell types across all three culture conditions. Scale bar: 50 μm.

Mixed co-culture suppresses migration of both ECs and SMCs

ECs and SMCs were cultured and observed in real time under mono-culture, non-contact co-culture, and mixed co-culture conditions 12 h after insert removal. Representative fluorescence images are shown in Figure 3A. The detailed experimental workflow is schematically illustrated in Figure S3, and representative stitched fluorescence micrographs of the global field of view are provided in Figure S4. In mono-culture, both ECs and SMCs migrated collectively toward the cell-free region, exhibiting relatively far frontiers. Under non-contact co-culture conditions, the frontiers of both cell types were reduced, and mixed co-culture led to a further decrease of migration distance.

The median migration distance was used to represent the position of the collective migration distance, as illustrated in Figure 3B. Quantitative analysis based on the mid-cell migration showed a graded decrease in collective migration speed from mono-culture to non-contact co-culture and to mixed co-culture, as shown in Figure 3C. This suppressive effect was observed for both cell types; however, the extent of suppression differed between the two cell types. ECs exhibited a more pronounced reduction in migration velocity under co-culture conditions, particularly mixed co-culture, whereas SMCs showed a comparatively moderate decrease.

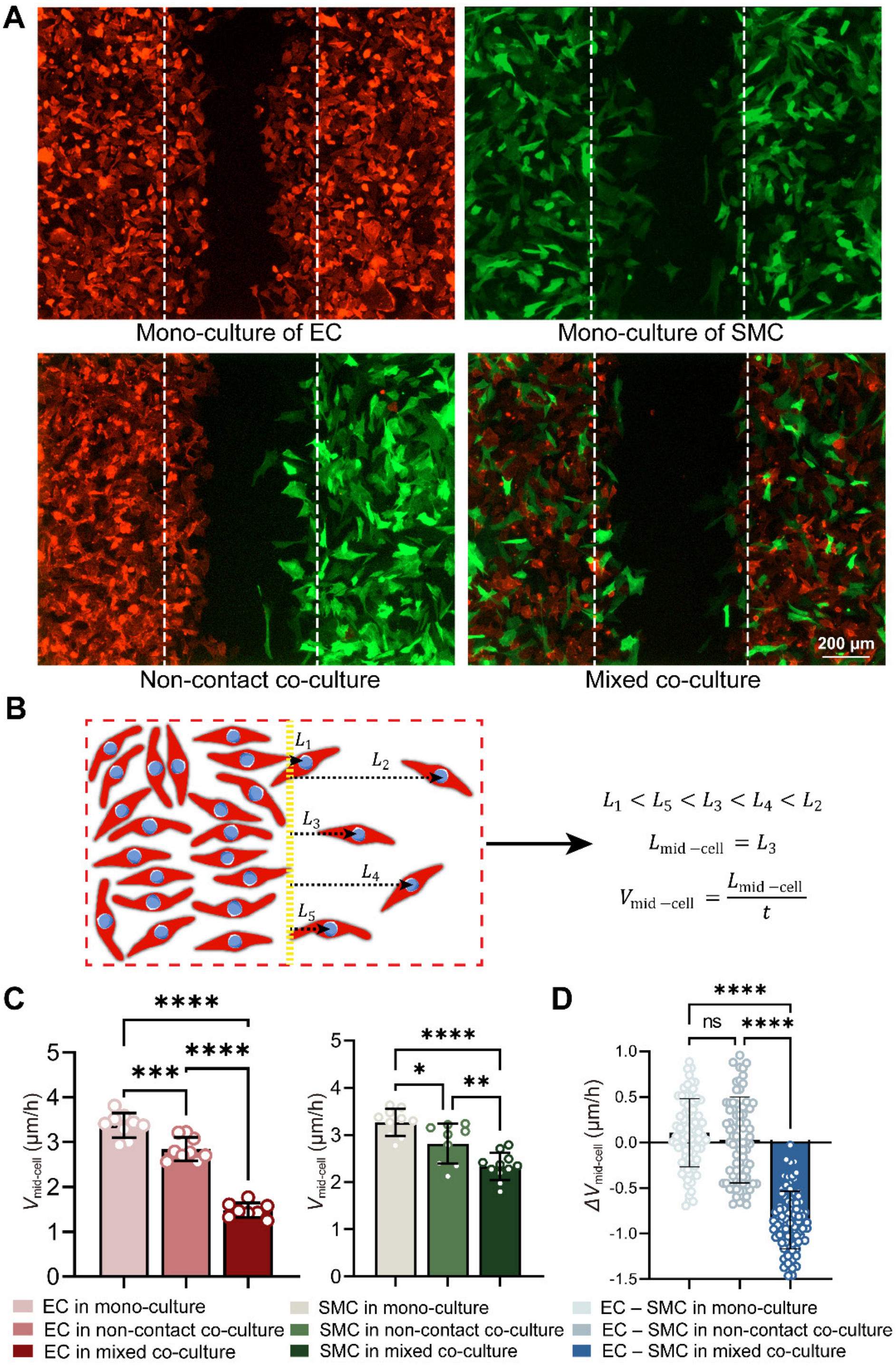

Figure 3. Collective cell migration of ECs (actin in red) and SMCs (actin in green) on cell culture plate under the indicated three distinct culture conditions reflecting that co-culture slows down cell migration and mixing even more. (**A**) Representative fluorescence images showing collective migration of ECs and SMCs 12 h after removal of the inserts. White dashed lines indicate the initial wound edges. Clear differences in collective migration patterns are observed among different culture conditions. In non-contact co-culture, collective cell migration is reduced; in mixed co-culture, collective migration is further suppressed. (**B**) Schematic illustration of the $V_{\text{mid-cell}}$ measurement. Distances from the initial wound edge to the nuclei of the leading cells (L_1–L_5) are extracted, and the median distance (L_3 in this example) is used to represent the

collective migration distance. This metric minimizes the influence of outlier cells and more reliably reflects coordinated collective cell migration. (**C**) Quantitative evaluation of the mid-cell migration velocity, V(mid-cell), reflecting the collective cell migration velocity for ECs and SMCs under different culture conditions ($n = 5$). (**D**) Quantitative comparison of the difference in collective migration velocity between ECs and SMCs under different culture conditions. In mono-culture and non-contact co-culture, ECs migrate faster than or similar to SMCs. However, there is a reversal in direct contact, with SMCs surpassing ECs in collective migration. Error bars represent mean ± standard deviation (s.d.). Statistical significance is indicated as $^{*}p < 0.05$, $^{**}p < 0.01$, $^{***}p < 0.001$, and $^{****}p < 0.0001$.

Random migration behaviors of ECs and SMCs also differ across mono-culture, non-contact co-culture, and mixed co-culture conditions, as shown in Figure 4A. Individual cell tracking revealed progressively shorter and more confined migration trajectories under co-culture conditions for both cell types, with the most restricted paths observed in mixed co-culture, as shown in Figure 4B. Random migration was quantified using the contour velocity and diffusion coefficient derived from single-cell trajectories, as illustrated in Figure 4C. Quantitative analysis showed a reduction in V_{contour} from mono-culture to mixed co-culture for both ECs and SMCs, as shown in Figure 4D. A similar trend was observed for the diffusion coefficient (D), with a pronounced suppression observed in ECs compared with SMCs.

Together, these results indicate that co-culture exerts an inhibitory effect on cell migration, with increasing suppression under conditions allowing greater intercellular interaction. Importantly, CCK-8 assays showed no significant differences in cell viability across culture conditions, as shown in Figure S5, excluding the possibility that the reduced migration resulted from compromised cell survival or metabolic activity. Furthermore, similar suppression of migration was consistently observed across a range of seeding densities, as shown in Figure S6, demonstrating that this inhibitory effect was robust and the basic trend was not altered with initial cell densities.

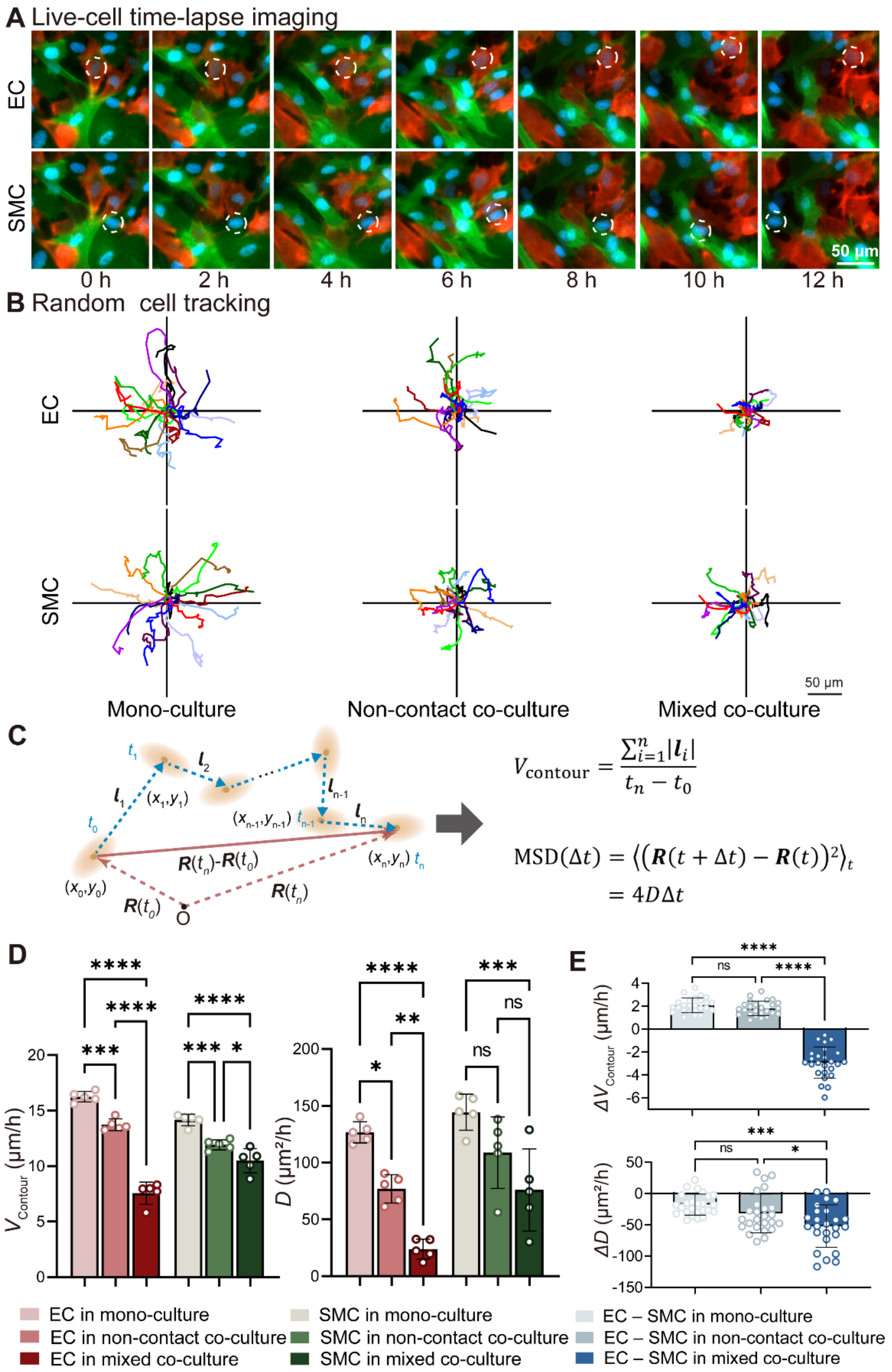

Figure 4. Random migration of ECs and SMCs on cell culture plates under different culture conditions. (**A**) Live-cell time-lapse imaging of ECs (red) and SMCs (green), with nuclei stained in blue, recorded over 12 h under different culture conditions at a density of 3 × 10^5 cells/mL. The observation started at 8 h after cell culturing. Representative cells tracked during migration are indicated by white dashed circles. Scale bar, 50 μm. (**B**) Cell random tracking plots showing migration trajectories of

individual cells under the indicated conditions. Each line represents the path of a single cell, with all tracks normalized to a common origin to facilitate comparison of migration behaviors. Scale bar, 50 μm. Top Row: ECs in mono-culture, non-contact co-culture and mixed co-culture, and bottom row: SMCs in mono-culture, non-contact co-culture and mixed co-culture. Each sample tracked at least 15 cells per trial (n = 5 for each group). (**C**) Schematic illustration of the calculations of cell migration contour velocity ($V_{contour}$) and diffusion coefficient (D). The $V_{contour}$ is obtained by summing the stepwise displacements $|\boldsymbol{l}_i|$ along the migration trajectory and dividing by the total observation time $(t_i - t_0)$, representing the actual path length traveled by the cell per unit time. D is derived from the mean squared displacement (MSD). (**D**) Quantitative analysis of $V_{contour}$ and D across different groups. Bar graphs show mean ± s.d., and the statistical comparison highlights significant differences in migration dynamics among culture conditions. Contour velocity $V_{contour}$ is derived from the contour length of the trajectory over the total time, representing the path taken by the cell. The diffusivity, based on MSD, remained independent of the time interval. (**E**) Quantitative comparison of the relative migration between ECs and SMCs under different culture conditions. $\Delta V_{contour}$ and ΔD represent the differences in contour velocity and diffusion coefficient, respectively. Bar graphs show mean ± s.d. Statistical significance is indicated as $*p < 0.05$, $**p < 0.01$, $***p < 0.001$, and $****p < 0.0001$.

Distance-dependent effect of paracrine signaling and increased surface coverage from physical mixing underlie the reduced cell migration in mixed co-culture

After analysis, we found that the effects observed in mixed co-culture may be explained by two factors: (1) decrease of the effect in paracrine signaling with intercellular distance, and (2) increased coverage resulting from physical mixing, as schematically presented in Figure 5A. While the former is apparent according to comparison between the groups of mixed co-culture and transwell co-culture in Figures 3 and 4, the latter is presented in Figure 5. Fluorescence imaging showed an increase in cell coverage under mixed co-culture compared with mono-culture conditions for both ECs and SMCs, as shown in Figure 5B. In mono-culture, ECs and SMCs exhibited surface coverages of approximately 30% and 34%, respectively, whereas mixed co-culture increased the coverage to about 45%. Quantitative analysis further revealed that cells in mixed co-culture displayed significantly larger spreading areas than those in mono-culture for both cell types, as reflected by the increased mean spreading area in the bar plots.

To assess how cell spreading area changes over time, five representative cells were selected and tracked during the 12 h migration period, as shown in Figure 5C (left). Although the spreading area of individual cells fluctuated over time, its overall level remained relatively stable within the observation period, permitting the use of a time-averaged spreading area to represent the spreading area of each cell. The time-averaged spreading area of each cell was calculated and listed together with the corresponding D in Figure 5C (right). These values were used for the subsequent analysis of the relationship between cell spreading area and D. For both ECs and SMCs, the relationship between cell spreading area and D was examined, as shown in Figure 5D. In each cell type, larger spreading areas were associated with lower diffusion coefficients, indicating a consistent negative correlation between cell spreading and migration speed.

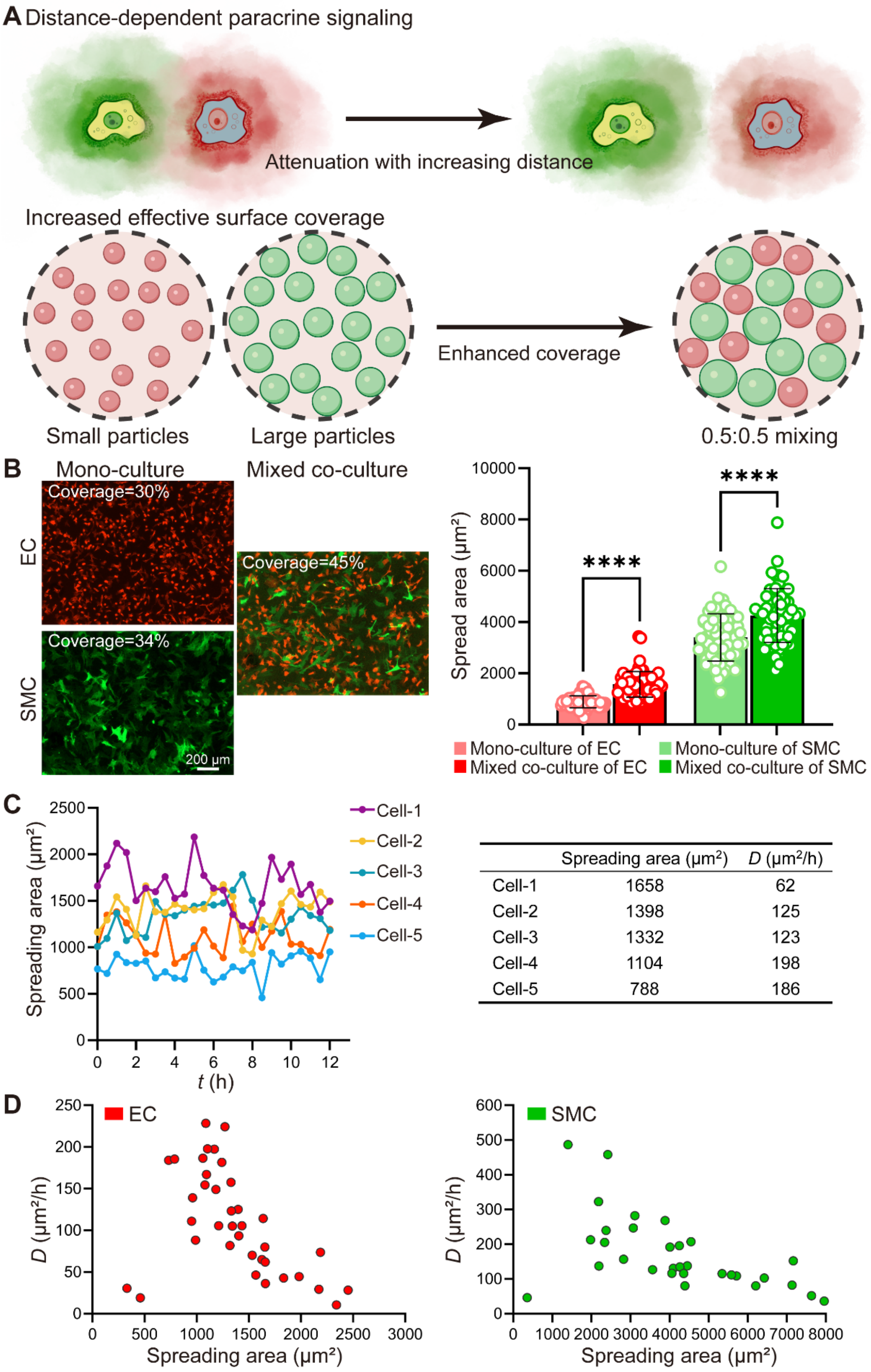

	Spreading area (μm²)	D (μm²/h)
Cell-1	1658	62
Cell-2	1398	125
Cell-3	1332	123
Cell-4	1104	198
Cell-5	788	186

Figure 5. Synergistic effects of putative distance-dependent paracrine signaling and enhanced effective surface coverage in mixed co-culture conditions. (**A**) Schematic illustration of the two proposed concurrent mechanisms potentially underlying the

mixed co-culture effect. Upper panel: the model depicts that paracrine signaling between heterotypic cells is distance-dependent and becomes attenuated with increasing intercellular separation, highlighting the importance of close spatial proximity for effective paracrine communication. Lower panel: from a geometric perspective, the schematic illustrates that compared with monodisperse assemblies of small or large particles, a 0.5:0.5 mixture of small and large particles allows smaller particles to occupy interstitial voids between larger ones, thereby increasing the effective surface coverage and the total accessible area. (**B**) Representative fluorescence images of endothelial cells (ECs, red) and smooth muscle cells (SMCs, green) cultured in mono-culture and mixed co-culture conditions at indicated time points (12 h). Cells in mixed co-culture exhibit enhanced coverage compared with those in mono-culture conditions. Bar graphs show mean ± s.d. ****$p < 0.0001$. Scale bar: 200 μm. (**C**) Time-dependent changes in the spreading area of five representative cells over 12 h adhesion. For each cell, the spreading area was averaged over time to obtain its mean spreading area for subsequent analysis. The corresponding mean spreading area and diffusion coefficient (D) are listed in the table on the right, with cells ranked in descending order of spreading area. (**D**) Statistical analysis of the relationship between cell spreading area and diffusion coefficient for ECs and SMCs. A significant negative correlation is observed for both cell types, indicating that cells with larger spreading areas exhibit lower diffusion coefficients.

Fabrication of RGD nanopatterns with tunable nanospacing

RGD nanopatterns with tunable nanoscale ligand spacing were fabricated using block copolymer micelle nanolithography, as schematically illustrated in Figure 6A. Representative SEM images showed highly ordered Au nanopatterns with uniform nanodot distribution, as shown in Figure 6B. By adjusting the molecular weight of the PS-*b*-P2VP block copolymers and the dip-coating parameters, the nanospacing between neighboring nanodots was systematically tuned over a wide range, from approximately 29 to 121 nm. For each nanospacing, the nanopatterns exhibited a high degree of regularity and spatial order, with narrow spacing distributions, indicating good reproducibility of the fabrication process. The corresponding fabrication parameters and resulting nanospacings are summarized in Figure 6B, demonstrating that controlled variation of polymer characteristics and dip-coating conditions allow precise and predictable modulation of ligand nanospacing. Together, these results confirm the successful preparation of a nanopatterning platform that provides well-defined and adjustable RGD nanospacing for subsequent investigations of cell–cell interactions on biomaterial surfaces.

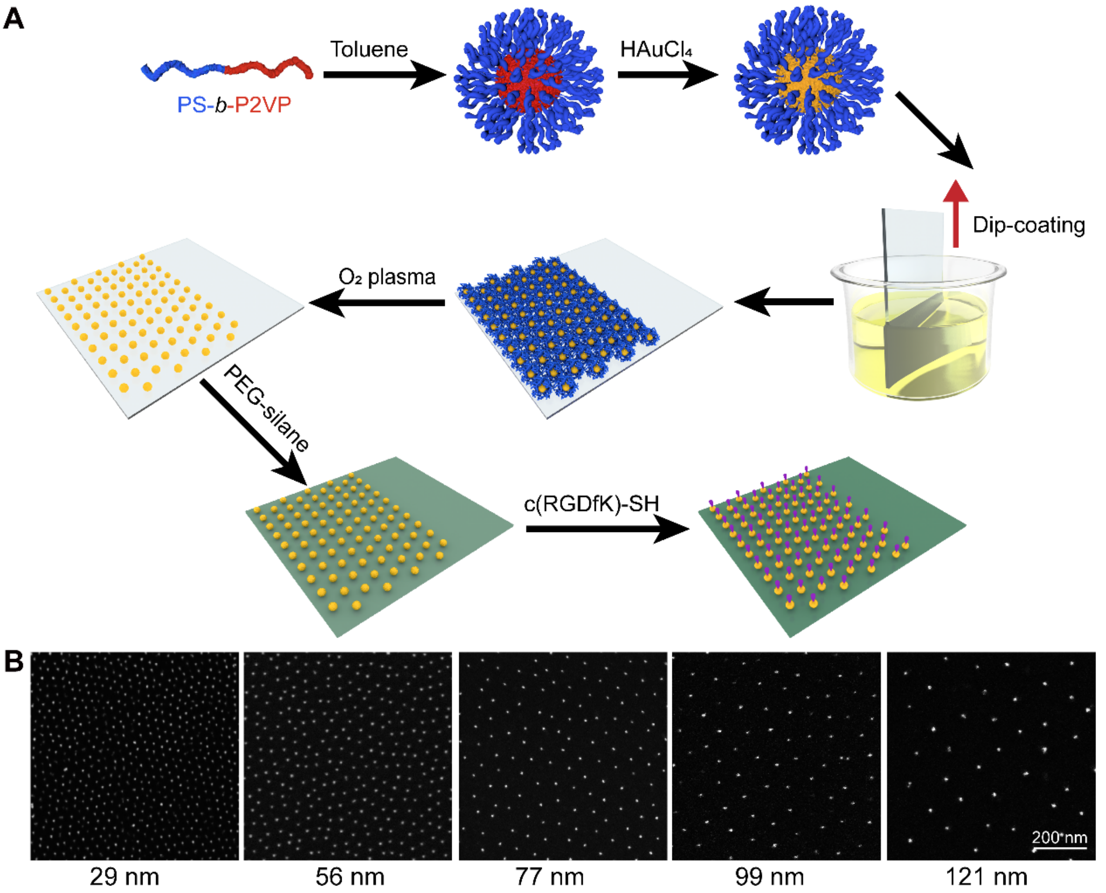

Block copolymer	M_n	Concentration/ mg·mL^{-1}	Loading amount	Pulling speed/ µm·s^{-1}	Nanospacing/ nm
P18225	33300-*b*-11000	5.0	0.50	300	29 ± 1
P4633	79000-*b*-36500	5.0	0.60	400	56 ± 4
P3021	148500-*b*-19000	5.0	0.60	400	77 ± 5
P5052	185000-*b*-32000	2.0	0.50	400	99 ± 3
P5052	185000-*b*-32000	2.0	0.50	200	121 ± 5

Figure 6. Fabrication and functionalization of RGD nanopatterns with tunable nanospacing. (**A**) Schematic illustration of block copolymer micelle nanolithography technique for preparing ordered RGD nanopatterns with nonfouling background. (**B**) Representative SEM images of Au nanopatterns with different nanospacings, demonstrating a high degree of order and precise nanoscale control over ligand spacing, together with a summary of the fabrication parameters used to achieve the corresponding nanospacings. The nanopatterns were prepared using poly(styrene-*block*-2-vinylpyridine) (PS-*b*-P2VP) block copolymers with different molecular weights (M_n). The polymer concentration refers to the concentration of PS-*b*-P2VP in toluene, and the loading amount is defined as the molar ratio of $HAuCl_4 \cdot 3H_2O$ to 2-vinylpyridine units in the block copolymer micelles. The pulling speed corresponds to the substrate retracting velocity during dip-coating. Nanospacing represents the lateral spacing of Au nanodots on glass substrates and is reported as mean ± s.d. Scale bar: 200 nm.

RGD nanospacing modulates adhesion and relative migration of ECs and SMCs in mixed co-culture

To enable controlled modulation of migration behaviors arising from EC–SMC interactions, RGD nanopattern surfaces with defined ligand nanospacing were employed as a biomaterial platform. Characterization of cell adhesion on these nanopatterns revealed a spacing-dependent reduction in both adhesion number and spreading area for ECs and SMCs, confirming effective modulation of cell–substrate interactions, as shown in Figure 7.

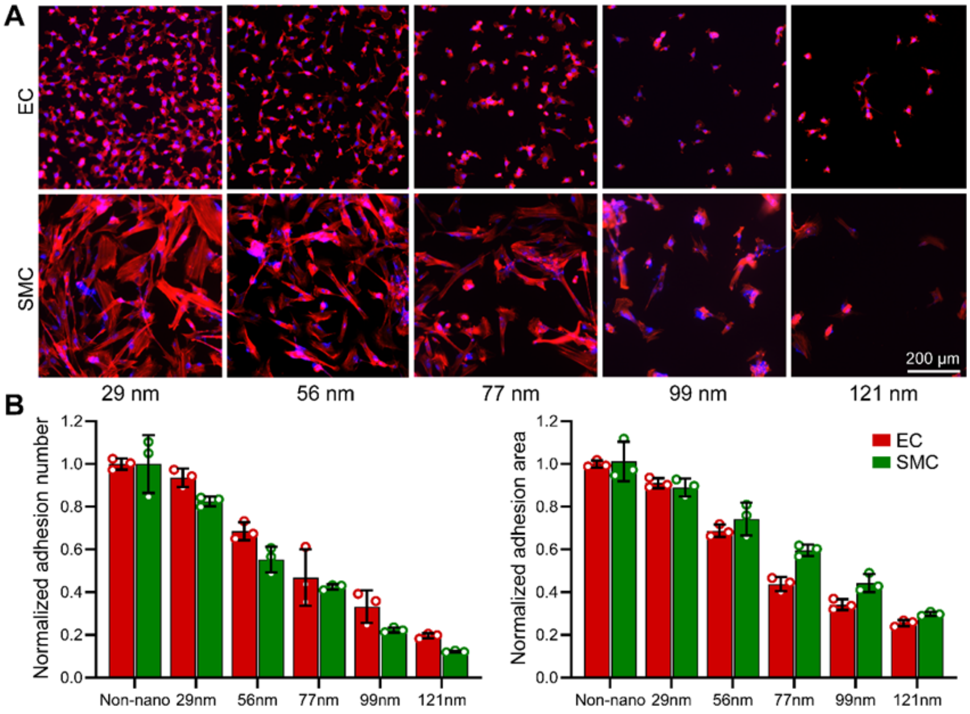

Figure 7. Adhesion behavior of ECs and SMCs on nanopatterns of different nanospacings. (**A**) Representative immunofluorescence images of ECs and SMCs cultured on nanopatterns of different RGD nanospacings. F-actin was stained in red and nuclei in blue. Both cell types exhibited progressively reduced adhesion and spreading area as the ligand nanospacing increased. Scale bar: 200 µm. (**B**) Quantitative analysis of normalized adhesion number (left) and spreading area (right). ECs and SMCs both showed a spacing-dependent decrease in adhesion. Statistical analysis was performed separately for each cell type using one-way ANOVA. For normalized adhesion number, significant differences among nanospacing groups were observed for ECs (global $p = 1.98 \times 10^{-8}$) and SMCs (global $p = 3.96 \times 10^{-9}$). For normalized spreading area, significant differences among nanospacing groups were also observed for ECs (global $p = 2.41 \times 10^{-13}$) and SMCs (global $p = 1.86 \times 10^{-8}$). Data are presented as mean ± s.d.

Fluorescence imaging revealed a clear contrast between cell-adhesive and non-adhesive regions on the nanopattern surfaces, as shown in Figure 8A. To first examine whether the mixed co-culture-induced suppression of migration rate observed on culture plates is preserved on nanopatterns, collective migration of ECs and SMCs was evaluated using a scratch-wound assay on a representative RGD nanopattern with a nanospacing of 29 nm, as shown in Figure 8B. On nanopatterned surfaces, both ECs

and SMCs migrated toward the wound region under mono-culture conditions, whereas mixed co-culture resulted in a clear reduction in migration rate for both cell types. Quantitative analysis of the $V_{\text{mid-cell}}$ confirmed that the suppressive effect of mixed co-culture remained evident on the nanopattern surface.

In the experiments presented above, both random and collective migration showed a consistent suppressive effect of mixed co-culture on cell migration. Moreover, collective migration better reflects coordinated cell movement relevant to vascular repair. Quantitative analysis of random migration requires tracking individual trajectories; typically, at least 15 cells were analyzed per sample with positions recorded at 25 time points, yielding approximately 375 coordinate data points. These data were further used to calculate MSD and D, involving additional computational processing and fitting. In contrast, collective migration can be directly evaluated from wound front displacement with substantially fewer measurements, and was therefore used to assess the effect of nanopatterns on co-culture migration behavior.

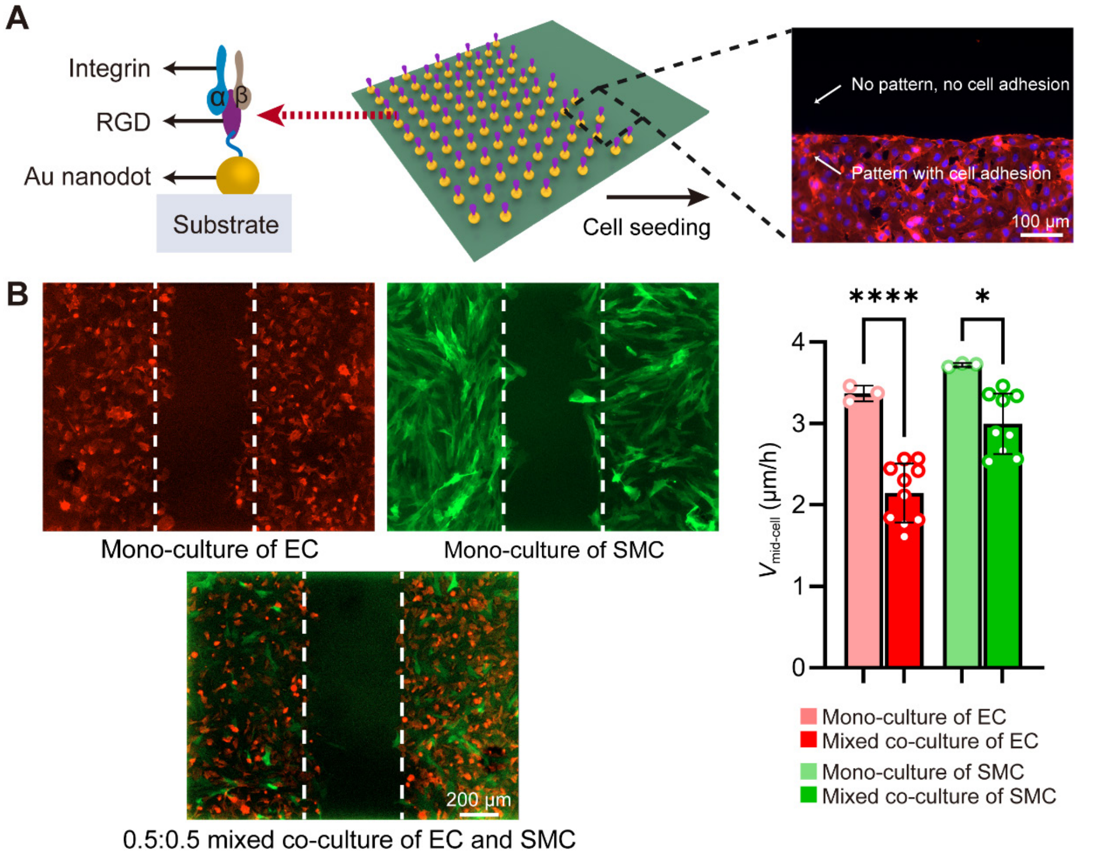

Figure 8. Collective migration of EC and SMC on nanopatterned surfaces under different culture conditions. (A) Schematic of integrin-RGD engagement on nanopattern surface and its relevance to cell adhesion. RGD ligands immobilized on Au nanodots specifically bind integrins, enabling nanoscale regulation of cell adhesion and organization. The fluorescence image illustrates cellular responses on the nanopatterned surfaces, showing a clear dipline and adhesive contrast. Scale bar: 100 μm. (**B**) Collective migration of endothelial cells (ECs, red) and smooth muscle cells (SMCs, green) on nanopatterns of 29 nm nanospacing. Fluorescence micrographs (top) show cell migration over 12 h, with dashed lines indicating initial wound boundaries in the scratch-wound assay. The bar graph (bottom) quantitatively compares mid-cell migration velocities of ECs and SMCs under mono-culture and mixed co-culture

(0.5:0.5 ratio) conditions. Results confirm that the previously observed decrease in migration during later stages of mixed co-culture on 29 nm nanopatterns. Scale bar: 200 µm. Statistical significance is indicated as $*p < 0.05$ and $****p < 0.0001$.

Having established that the suppression of collective migration is preserved on nanopattern, we next examined how ligand nanospacing modulates the relative migration of mixed co-cultured ECs and SMCs. To this end, collective migration was compared across RGD nanopatterns with varied nanospacings (Figure 9A). Fluorescence imaging revealed clear differences in collective migration across the nanopatterns, indicating a pronounced dependence on ligand nanospacing. Quantitative analysis showed that both ECs and SMCs exhibited nanospacing-dependent collective migration rate, with the highest migration rates observed on nanopatterns with 77 nm of RGD nanospacing. In contrast, migration was reduced on both smaller and larger nanospacings (Figure 9B), demonstrating a non-monotonic dependence of collective migration on nanoscale ligand organization in mixed co-culture condition.

Beyond absolute migration rate, the relative migration behavior between ECs and SMCs was also strongly influenced by RGD nanospacing. Analysis of the pairwise differences in $V_{\text{mid-cell}}$ revealed systematic shifts in the migration rate between the two cell types across different nanospacings, as shown in Figure 9C. At smaller nanospacings, ECs exhibited lower migration velocities than SMCs, whereas increasing the ligand spacing progressively reduced this difference and, at larger nanospacings, led to a reversal in the relative migration rates, with ECs migrating faster than SMCs. CCK-8 measurements revealed a gradual decrease in cell viability with increasing ligand nanospacing (Figure S7), indicating that cell migration on nanopatterned substrates is influenced by both co-culture interactions and ligand nanospacing. Together, these results illustrate that the nanoscale control of ligand nanospacing provides an effective means to tune not only the overall level of collective migration, but also the relative migratory behaviors of ECs and SMCs under co-culture conditions.

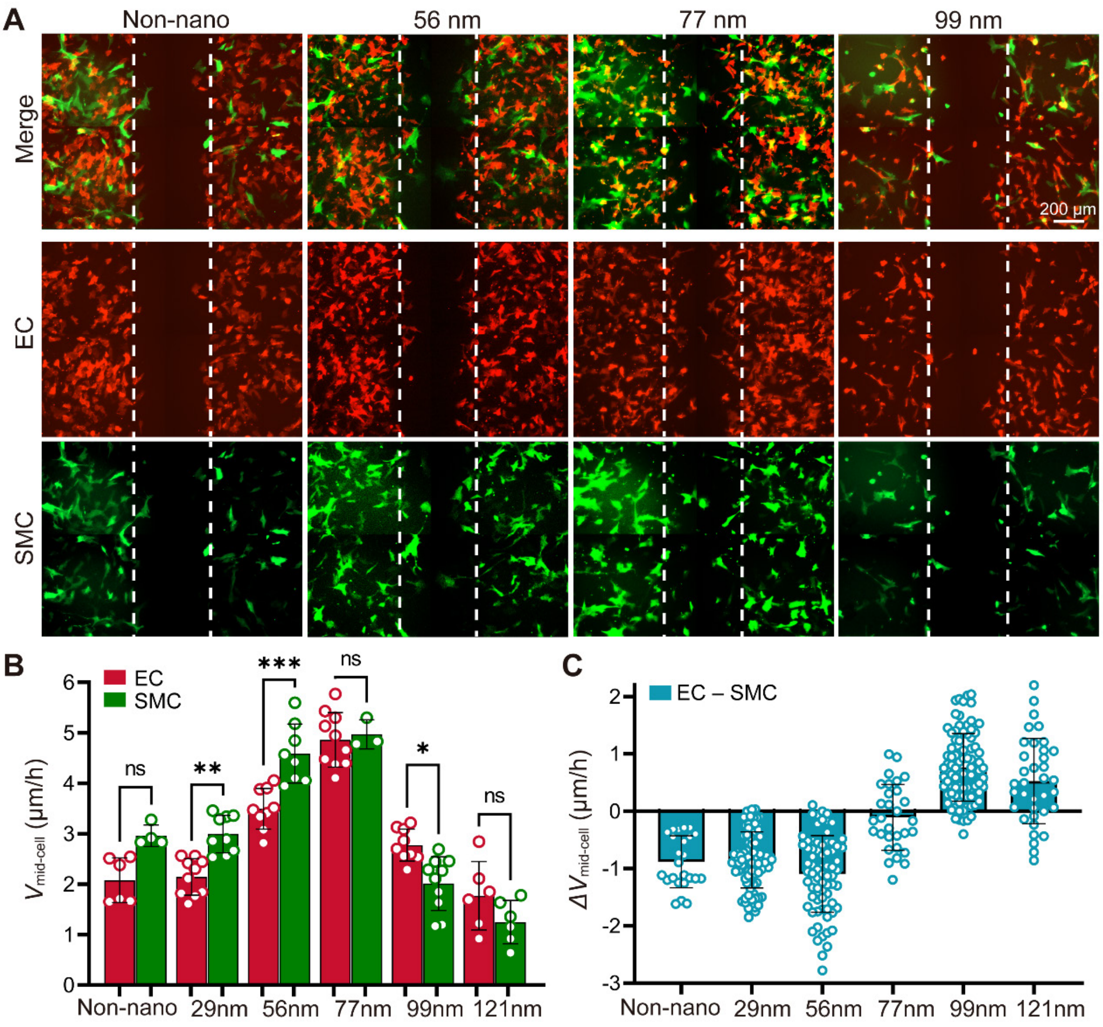

Figure 9. Effects of RGD nanospacings on collective migration of co-cultured ECs and SMCs. (**A**) Representative fluorescence micrographs of co-cultured ECs (red) and SMCs (green) on nanopatterns of different nanospacing after culturing for 12 h. For each nanospacing, the representative image was acquired from a single sample using a 2 × 2 tiled scan to provide a larger field of view while maintaining relatively high image resolution. For each condition, the merged view (top), EC-specific channel-RFP (middle), and SMC-specific channel-GFP (bottom) are presented. Collective migration was evaluated using a scratch-wound assay, with white dashed lines marking the initial wound boundaries. Scale bar: 200 μm. (**B**) Quantitative analysis of collective migration velocity, $V_{\text{mid-cell}}$, of ECs and SMCs under mixed co-culture conditions across different RGD nanopattern spacings. Distinct nanospacing-dependent migratory behaviors were observed for the two cell types: ECs and SMCs exhibited the highest migration rate on 77-nm RGD nanopatterns. Data are presented as mean ± s.d. Statistical significance is indicated as *$p < 0.05$, **$p < 0.01$, and ***$p < 0.001$. ns, not significant. (**C**) Quantitative analysis of the relative migration velocity between ECs and SMCs across different nanopattern spacings, calculated as the pairwise differences between their corresponding migration velocities (ECs minus SMCs). Data points in the histogram represent all possible combinations of the minuends and subtrahends derived from the individual measurements. The overall statistical significance among all groups was evaluated by one-way ANOVA, resulting in $p = 5.89 \times 10^{-72}$, which illustrates the dependence of the relative migration of the two cell types upon RGD nanospacing on the nanopatterned substrate.

Discussion

Paracrine signaling and physical mixing jointly contribute to reduced migration in mixed EC–SMC co-culture

Vascular injury or endothelial disruption has been widely reported to activate EC and SMC migration and to promote neointimal remodeling [38]. However, it remains elusive how ECs and SMCs influence each other's migration in mixed co-culture. Our present study has demonstrated that the mixed co-culture of ECs and SMCs resulted in a more pronounced reduction in cell migration than the non-contact co-culture (transwell). Although both co-culture configurations allow paracrine communication compared to mono-culture, direct mixing markedly shortens intercellular distances and increases heterotypic interfaces. In addition, because mixed co-culture brings the two cell types into direct contact, contact-dependent juxtacrine signaling may also contribute to the observed effect. While fluid dynamics in culture dishes differ from in vivo tissue distribution, the proximity in the mixed system conceptually mimics the physiological cell-cell adjacency. This spatial arrangement may facilitate higher local effective concentrations of secreted factors and altered paracrine signaling dynamics compared to the diluted environment of the transwell system [39-42]. Such co-culture mode has also been reported to activate additional signaling pathways that are not engaged under non-contact co-culture, thereby potentially reshaping the paracrine protein repertoire [43, 44]. Previous studies have shown that endothelial cells and smooth muscle cells form an intercellular signaling network whose integrated output contributes to the maintenance of vascular stability under homeostatic conditions [45-47]. Beyond TGF-β–mediated negative feedback [48, 49], direct EC-SMC contact enables multiple juxtacrine or proximity-restricted signaling axes, including Jagged–Notch [45, 50-52], ephrinB2 [53], N-cadherin–mediated adhesion signaling [54], and myoendothelial gap-junction communication [55], all of which have been implicated in restraining cell migration and reinforcing vessel wall organization. Consistent with these prior observations, the more pronounced migration suppression observed in mixed EC–SMC co-culture in our study is likely attributable, at least in part, to the enhanced engagement of these contact- and proximity-dependent signaling networks, which collectively bias EC–SMC crosstalk toward a vessel-stabilizing, migration-restraining state.

In addition to chemical signaling, physical mixing is likely to contribute to the observed reduction in migration as well. From a geometrical perspective, mixing of two cell types with distinct sizes and migration behaviors allows smaller cells to occupy interstitial spaces between larger cells, thereby increasing the surface coverage [56, 57]. This heterogeneous packing geometry increases the prevalence of short-range cell–cell interactions and elevates effective surface coverage, together imposing a physical constraint on cell migration. Because HUVECs and HASMCs differ in size and spreading behavior, the same initial cell number density does not necessarily correspond to the same effective spatial crowding. Consistent with this interpretation, cell spreading area is often inversely correlated with migration speed, a trend also observed in our system, as shown in Figure 5D. Together, our findings suggest that the enhanced migration suppression observed in mixed EC–SMC co-culture reflects the combined influence of putative proximity-enhanced paracrine signaling, contact-dependent juxtacrine signaling responses, and physical constraints arising from increased coverage. While our current data delineate the functional impact of this co-

culture mode, further investigations are warranted to dissect the specific molecular signaling programs and quantify their precise contributions.

Nanopatterns as a biomaterial strategy to control relative migration of ECs and SMCs

EC–SMC interactions in co-culture can reshape the relative migration of the two cell types. We further address whether such relative migration can be externally intervened and deliberately controlled, rather than being passively modulated by cell–cell interactions alone. Biomaterial techniques can be employed to regulate cells and cell-material interactions [58-62], and surface nanopatterning has emerged as an effective and tunable strategy to regulate cell behaviors [36]. In our previous work, we have demonstrated that ECs and SMCs exhibit distinct migratory sensitivities to nanoscale ligand spacing under mono-culture conditions [63]. These findings established nanopatterns as a potent regulator of single-cell migration. Nevertheless, it remains unclear whether such differential sensitivity can be translated into controllable relative migration behaviors under co-culture conditions.

Here, by examining EC–SMC co-culture on a series of nanoarrays, we uncover a distinct response pattern that differs from mono-culture behavior. As shown in Figure 9, both ECs and SMCs reached their maximal migration velocity at an intermediate RGD nanospacing of 77 nm. Analysis of relative migration reveals that nanospacing actively biases the migration balance between ECs and SMCs. SMCs exhibit a clear migratory advantage at smaller nanospacings, whereas increasing the spacing beyond ~70 nm favors EC migration. At small nanospacings (<70 nm), closely spaced ligands support stable focal adhesion formation and extensive cell spreading. Under this condition, increasing nanospacing is expected to facilitate focal adhesion turnover by moderately weakening adhesion stability, thereby promoting migration. In contrast, at larger nanospacings (>70 nm), widely spaced ligands impair the formation of nascent adhesions, leading to reduced spreading, weakened cell–cell interactions, and ultimately diminished migration. Various biochemical and biophysical strategies have been used to modulate the relative migration of EC and SMC, including soluble factors [64], chemotactic gradients [65], substrate stiffness [66], surface topography [67], and surface chemical modification [68, 69]. Here, we demonstrate that ligand nanospacing provides a distinct means to regulate relative migration under co-culture conditions by selectively amplifying, attenuating, or even reversing the migratory advantage of one cell type over the other. By precisely controlling ligand nanospacing without introducing exogenous biochemical signals, nanopattern provides a stable biomaterial platform to bias competitive, cell-type-specific migration under co-culture conditions. These findings point to potential applications in the design of biomaterial interfaces that require coordinated organization of multiple cell types, such as the surface design of tissue-engineering scaffolds and regenerative biomaterials. At the same time, relative migration should be considered together with cell viability. Larger nanospacings shifted the migration balance toward endothelial cells while being associated with reduced viability, highlighting the need to optimize nanospacing by balancing migration selectivity with cytocompatibility for endothelialization-related applications.

Our study utilized a two-dimensional culture configuration to enable precise control over ligand nanospacing and facilitate systematic analysis of relative migration behaviors. While this simplified model allows for high-resolution analysis, it does not fully recapitulate the three-dimensional architecture, matrix confinement, and mechanical heterogeneity encountered by cells in native tissues [70-72]. In addition, the endothelial cells and smooth muscle cells used in this study were derived from

different vascular sources. Although HUVECs were selected as a widely used and experimentally robust endothelial cell model for the migration assays used here, differences in vascular origin with HASMCs may influence the baseline migratory and mechanobiological properties of the two cell types. Future studies using matched-source endothelial and smooth muscle cells, as well as three-dimensional or multilayered culture systems, will help evaluate the generality of the present findings under more physiologically relevant microenvironmental conditions.

Conclusion

The migration behaviors of endothelial and smooth muscle cells have been analyzed under both mono-culture and mixed co-culture conditions. Direct mixed co-culture markedly suppressed the migration of both cell types while amplifying the migratory dominance of SMCs over ECs. We attribute this phenomenon primarily to enhanced intercellular interactions facilitated by reduced cell–cell spacing and increased effective surface coverage following physical mixing. Furthermore, by precisely controlling RGD nanospacing, we demonstrate that integrin-mediated adhesion at the nanoscale provides an effective means to tune multicellular migration dynamics, enabling not only modulation of absolute migration rates but also the relative migration between ECs and SMCs. Together, these findings indicate that relative cell migration in a system of multiple cell types emerges from the coupled regulation of cell–cell interactions and cell–substrate adhesion, providing a strategy to guide cell organization on biomaterials. This study establishes a rational basis for designing regenerative biomaterials that support functional endothelialization while maintaining controlled SMC migration, with implications for the development of next-generation vascular implants and tissue-engineered constructs.

Funding

This study was supported by National Natural Science Foundation of China (grant No. 52130302), and National Key R&D Program of China (grant No. 2023YFC2410300).

Supporting data

Supporting information includes 7 figures and 2 videos.

Conflicts of interest statement.

There are no conflicts to declare.

References

1. Basatemur GL, Jorgensen HF, Clarke MCH, Bennett MR, Mallat Z. Vascular smooth muscle cells in atherosclerosis. *Nat Rev Cardiol* 2019;16:727-744.
2. Grodecki K, Geers J, Kwiecinski J, Lin AD, Slipczuk L, Slomka PJ, Dweck MR, Nerlekar N, Williams MC, Berman D, Marwick T, Newby DE, Dey D. Phenotyping atherosclerotic plaque and perivascular adipose tissue: signalling pathways and clinical biomarkers in atherosclerosis. *Nat Rev Cardiol* 2025;22:443-455.
3. Casscells W. Migration of smooth muscle and endothelial cells. Critical events in restenosis. *Circulation* 1992;86:723-9.
4. Gong S, Li Y, Yan K, Shi Z, Leng J, Bao Y, Ning K. The Crosstalk Between Endothelial Cells, Smooth Muscle Cells, and Macrophages in Atherosclerosis. *Int J Mol Sci* 2025;26:1457.
5. García JR, García AJ. Biomaterial-mediated strategies targeting vascularization for bone repair. *Drug Deliv Transl Res* 2016;6:77-95.
6. Selvakumar PP, Rafuse MS, Johnson R, Tan W. Applying Principles of Regenerative Medicine to Vascular Stent Development. *Front Bioeng Biotechnol* 2022;10:826807.
7. Wang C, Lv J, Yang M, Fu Y, Wang W, Li X, Yang Z, Lu J. Recent advances in surface functionalization of cardiovascular stents. *Bioact Mater* 2025;44:389-410.
8. Shi D, Kang Y, Wang W, Liu R, Tang Q, Li Z, Jiang H, Ding J. Biodegradable polymeric occluder with controllable locking structure for closure of atrial septal defect via interventional treatment. *Regen Biomater* 2025;12:rbaf016.
9. Liu W, Wang Y, Wang J, Lanier OL, Wechsler ME, Peppas NA, Gu Z. Macroencapsulation Devices for Cell Therapy. *Engineering* 2022;13:53-70.
10. Wang G, Feng Y, Gao C, Zhang X, Wang Q, Zhang J, Zhang H, Wu Y, Li X, Wang L, Fu Y, Yu X, Zhang D, Liu J, Ding J. Biaxial stretching of polytetrafluoroethylene in industrial scale to fabricate medical ePTFE membrane with node-fibril microstructure. *Regen Biomater* 2023;10:rbad056.
11. Zhang W, Gao X, Zhang H, Sun G, Zhang G, Li X, Qi H, Guo J, Qin L, Shi D, Shi X, Li H, Zhang D, Guo W, Ding J. Maglev-fabricated long and biodegradable stent for interventional treatment of peripheral vessels. *Nat Commun* 2024;15:7903.
12. Zhang Y, Fu Y, Sun T, Li W, Yu L, Ding J. Skin Relevant Biomaterials from Wound Healing, Medical Aesthetics, Flexible Electronics to Artificial Intelligence and Beyond. *Adv Mater* 2025:e12919.
13. Müller MZ, Bernero M, Xie C, Qiu W, Oggianu E, Rabut L, Michaels TCT, Style RW, Müller R, Qin X-H. Cell-guiding microporous hydrogels by photopolymerization-induced phase separation. *Nat Commun* 2025;16:4923.
14. Tu C-X, Gao C-Y. Recent Advances on Surface-modified Biomaterials Promoting Selective Adhesion and Directional Migration of Cells. *Chin J Polym Sci* 2021;39:815-823.

15. Yang J, Wang P, Zhang Y, Zhang M, Sun Q, Chen H, Dong L, Chu Z, Xue B, Hoff WD, Zhao C, Wang W, Wei Q, Cao Y. Photo-tunable hydrogels reveal cellular sensing of rapid rigidity changes through the accumulation of mechanical signaling molecules. *Cell Stem Cell* 2025;32:121-136.
16. Liu W, Liu R, Chu LT, Wang X, Wu J, Ding J, Chen TH. Y-shaped DNA as a dynamic self-assembly nanomaterial for phenotype-specific regulation of stem cell differentiation on the gene level. *Regen Biomater* 2025;12:rbaf043.
17. Engler AJ, Sen S, Sweeney HL, Discher DE. Matrix Elasticity Directs Stem Cell Lineage Specification. *Cell* 2006;126:677-689.
18. Ye K, Wang X, Cao L, Li S, Li Z, Yu L, Ding J. Matrix stiffness and nanoscale spatial organization of cell-adhesive ligands direct stem cell fate. *Nano Lett* 2015;15:4720-4729.
19. Isomursu A, Park K-Y, Hou J, Cheng B, Mathieu M, Shamsan GA, Fuller B, Kasim J, Mahmoodi MM, Lu TJ, Genin GM, Xu F, Lin M, Distefano MD, Ivaska J, Odde DJ. Directed cell migration towards softer environments. *Nat Mater* 2022;21:1081-1090.
20. Yang J, Zhang Y, Wang S, Wang P, Dong L, Li L, Cheng Y, Huang X, Xue B, Wang W, Jiang C, Gu X, Cao Y, Wei Q. Dynamic rigidity changes enable rapid cell migration on soft substrates. *Nat Commun* 2025;16:8793.
21. Platzman I, Muth CA, Lee-Thedieck C, Pallarola D, Atanasova R, Louban I, Altrock E, Spatz JP. Surface properties of nanostructured bio-active interfaces: impacts of surface stiffness and topography on cell–surface interactions. *RSC Adv* 2013;3:13293-13303.
22. Liu D, Xiang T, Gong T, Tian T, Liu X, Zhou S. Bioinspired 3D Multilayered Shape Memory Scaffold with a Hierarchically Changeable Micropatterned Surface for Efficient Vascularization. *ACS Appl Mater Interfaces* 2017;9:19725-19735.
23. Xu X, Wang W, Liu Y, Bäckemo J, Heuchel M, Wang W, Nie Y, Iqbal I, Kratz K, Lendlein A, Ma N. Substrates mimicking the blastocyst geometry revert pluripotent stem cell to naivety. *Nat Mater* 2024;23:1748-1758.
24. Wang HY, Liu RL, Yu Y, Xue HR, Shen RJ, Zhang YS, Ding JD. Effects of cell shape and nucleus shape on epithelial-mesenchymal transition revealed using chimeric micropatterns. *Biomaterials* 2025;317:123013.
25. Shen Y, Gao M, Ma Y, Yu H, Cui F-z, Gregersen H, Yu Q, Wang G, Liu X. Effect of surface chemistry on the integrin induced pathway in regulating vascular endothelial cells migration. *Colloids Surf B Biointerfaces* 2015;126:188-197.
26. Shen Y, Zhang W, Xie Y, Li A, Wang X, Chen X, Liu Q, Wang Q, Zhang G, Liu Q, Liu J, Zhang D, Zhang Z, Ding J. Surface modification to enhance cell migration on biomaterials and its combination with 3D structural design of occluders to improve interventional treatment of heart diseases. *Biomaterials* 2021;279:121208.
27. Arnold M, Cavalcanti-Adam EA, Glass R, Blummel J, Eck W, Kantlehner M, Kessler H, Spatz JP. Activation of Integrin Function by Nanopatterned Adhesive Interfaces. *ChemPhysChem* 2004;5:383-388.

28. He J, Shen R, Liu Q, Zheng S, Wang X, Gao J, Wang Q, Huang J, Ding J. RGD Nanoarrays with Nanospacing Gradient Selectively Induce Orientation and Directed Migration of Endothelial and Smooth Muscle Cells. *ACS Appl Mater Interfaces* 2022;14:37436−37446.
29. Ammann KR, DeCook KJ, Tran PL, Merkle VM, Wong PK, Slepian MJ. Collective cell migration of smooth muscle and endothelial cells: impact of injury versus non-injury stimuli. *J Biol Eng* 2015;9:19.
30. Lee H-W, Shin JH, Simons M. Flow goes forward and cells step backward: endothelial migration. *Exp Mol Med* 2022;54:711-719.
31. Lavender MD, Pang Z, Wallace CS, Niklason LE, Truskey GA. A system for the direct co-culture of endothelium on smooth muscle cells. *Biomaterials* 2005;26:4642-4653.
32. Zheng X, Hu X, Zhang W. The phenotype of vascular smooth muscle cells co-cultured with endothelial cells is modulated by PDGFR-beta/IQGAP1 signaling in LPS-induced intravascular injury. *Int J Med Sci* 2019;16:1149-1156.
33. Li S, Wang X, Cao B, Ye K, Li Z, Ding J. Effects of nanoscale spatial arrangement of arginine-glycine-aspartate peptides on dedifferentiation of chondrocytes. *Nano Lett* 2015;15:7755-7765.
34. Liu X, Zhang M, Wang P, Zheng K, Wang X, Xie W, Pan X, Shen R, Liu R, Ding J, Wei Q. Nanoscale distribution of bioactive ligands on biomaterials regulates cell mechanosensing through translocation of actin into the nucleus. *Proc Natl Acad Sci USA* 2025;122:e2501264122.
35. Liu Q, Zheng S, Ye K, He J, Shen Y, Cui S, Huang J, Gu Y, Ding J. Cell migration regulated by RGD nanospacing and enhanced under moderate cell adhesion on biomaterials. *Biomaterials* 2020;263:120327.
36. Huang J, Grater SV, Corbellinl F, Rinck S, Bock E, Kemkemer R, Kessler H, Ding J, Spatz JP. Impact of order and disorder in RGD nanopatterns on cell adhesion. *Nano Lett* 2009;9:1111-1116.
37. Shen R, Zhang Z, Li J, Ding J. Nonmonotonic Dependence of Random Migration upon Density of Cells on Biomaterials. *ACS Appl Mater Interfaces* 2026;18: 12480-12493.
38. Khachigian LM, Lindner V, Williams AJ, Collins T. Egr-1-induced endothelial gene expression: a common theme in vascular injury. *Science* 1996;271:1427-31.
39. Heydarkhan-Hagvall S, Helenius G, Johansson BR, Li JY, Mattsson E, Risberg B. Co-culture of endothelial cells and smooth muscle cells affects gene expression of angiogenic factors. *J Cell Biochem* 2003;89:1250-1259.
40. Kook YM, Jeong Y, Lee K, Koh WG. Design of biomimetic cellular scaffolds for co-culture system and their application. *J Tissue Eng* 2017;8:2041731417724640.
41. Suzuki K, Chosa N, Sawada S, Takizawa N, Yaegashi T, Ishisaki A. Enhancement of Anti-Inflammatory and Osteogenic Abilities of Mesenchymal Stem Cells via Cell-to-Cell Adhesion to Periodontal Ligament-Derived Fibroblasts. *Stem Cells Int* 2017;2017:3296498.

42. Chen Y-C, Chang Y-W, Tan KP, Shen Y-S, Wang Y-H, Chang C-H. Can mesenchymal stem cells and their conditioned medium assist inflammatory chondrocytes recovery? *PLoS One* 2018;13:e0205563.
43. Zeng X, Yang P, Chen B, Jin X, Liu Y, Zhao X, Liang S. Quantitative secretome analysis reveals the interactions between epithelia and tumor cells by in vitro modulating colon cancer microenvironment. *J Proteomics* 2013;89:51-70.
44. Dietrich I, Girdlestone J, Giele H. Differential cytokine expression in direct and indirect co-culture of islets and mesenchymal stromal cells. *Cytokine* 2022;150:155779.
45. Lilly B. We Have Contact: Endothelial Cell-Smooth Muscle Cell Interactions. *Physiology* 2014;29:234-241.
46. Mendez-Barbero N, Gutierrez-Munoz C, Blanco-Colio LM. Cellular Crosstalk between Endothelial and Smooth Muscle Cells in Vascular Wall Remodeling. *Int J Mol Sci* 2021;22.
47. Sawasaki K, Nakamura M, Kimura N, Kawahito K, Yamazaki M, Fujie H, Sakamoto N. Endothelial-derived nitric oxide impacts vascular smooth muscle cell phenotypes under high wall shear stress condition. *Biochem Biophys Res Commun* 2024;740:151005.
48. Sato Y, Tsuboi R, Lyons R, Moses H, Rifkin DB. Characterization of the activation of latent TGF-beta by co-cultures of endothelial cells and pericytes or smooth muscle cells: a self-regulating system. *J Cell Biol* 1990;111:757-763.
49. Sato Y, Okada F, Abe M, Seguchi T, Kuwano M, Sato S, Furuya A, Hanai N, Tamaoki T. The mechanism for the activation of latent TGF-beta during co-culture of endothelial cells and smooth muscle cells: cell-type specific targeting of latent TGF-beta to smooth muscle cells. *J Cell Biol* 1993;123:1249-54.
50. Artavanis-Tsakonas S, Rand MD, Lake RJ. Notch signaling: Cell fate control and signal integration in development. *Science* 1999;284:770-776.
51. Xia Y, Bhattacharyya A, Roszell EE, Sandig M, Mequanint K. The role of endothelial cell-bound Jagged1 in Notch3-induced human coronary artery smooth muscle cell differentiation. *Biomaterials* 2012;33:2462-72.
52. Zohorsky K, Lin S, Mequanint K. Immobilization of Jagged1 Enhances Vascular Smooth Muscle Cells Maturation by Activating the Notch Pathway. *Cells* 2021;10:2089.
53. Korff T, Braun J, Pfaff D, Augustin HG, Hecker M. Role of ephrinB2 expression in endothelial cells during arteriogenesis: impact on smooth muscle cell migration and monocyte recruitment. *Blood* 2008;112:73-81.
54. Kruse K, Lee QS, Sun Y, Klomp J, Yang X, Huang F, Sun MY, Zhao S, Hong Z, Vogel SM, Shin JW, Leckband DE, Tai LM, Malik AB, Komarova YA. N-cadherin signaling via Trio assembles adherens junctions to restrict endothelial permeability. *J Cell Biol* 2019;218:299-316.
55. Straub AC, Zeigler AC, Isakson BE. The myoendothelial junction: connections that deliver the message. *Physiology (Bethesda)* 2014;29:242-9.

56. Brouwers HJH. Particle-size distribution and packing fraction of geometric random packings. *Phys Rev E* 2006;74:031309.
57. Chang CS, Chao J. A Review of Particle Packing Models and Their Applications to Characterize Properties of Sand-Silt Mixtures. *Geotechnics* 2024;4:1124-1139.
58. Wei Y, Ji Y, Xiao L-L, Lin Q-k, Xu J-p, Ren K-f, Ji J. Surface engineering of cardiovascular stent with endothelial cell selectivity for in vivo re-endothelialisation. *Biomaterials* 2013;34:2588-2599.
59. Bai L, Zhao J, Wang M, Feng Y, Ding J. Matrix-Metalloproteinase-Responsive Gene Delivery Surface for Enhanced in Situ Endothelialization. *ACS Appl Mater Interfaces* 2020;12:40121-40132.
60. Shi D, Kang Y, Jiang Z, Li X, Zhang H, Wang Q, Guo J, Jiang H, Luo Q, Ding J. Hybrid interpenetrating network of polyester coronary stent with tunable biodegradation and mechanical properties. *Biomaterials* 2024;304:122411.
61. Zhang Y, Sun T, Li W, Yu L, Ding J. Polymeric Skin Booster with Moderate Collagen Regeneration. *Adv Funct Mater* 2025:e14562.
62. Yu Y, Xue H, Wang H, Fu Y, Zhang Y, Yu W, Yu L, Wang J, Lu B, Ding J. A biomaterial-cell model of inflammation-induced neurodegeneration of Parkinson's disease. *Cell Biomater* 2026;2:100298.
63. He J, Liu Q, Zheng S, Shen R, Wang X, Gao J, Wang Q, Huang J, Ding J. Enlargement, reduction, and even reversal of relative migration speeds of endothelial and smooth muscle cells on biomaterials simply by adjusting RGD nanospacing. *ACS Appl Mater Interfaces* 2021;13:42344-42356.
64. Zhou J, Shao L, Yu J, Huang J, Feng Q. PDGF-BB promotes vascular smooth muscle cell migration by enhancing Pim-1 expression via inhibiting miR-214. *Ann Transl Med* 2021;9:1728.
65. Ferre-Torres J, Noguera-Monteagudo A, Lopez-Canosa A, Romero-Arias JR, Barrio R, Castaño O, Hernandez-Machado A. Modelling of chemotactic sprouting endothelial cells through an extracellular matrix. *Front Bioeng Biotechnol* 2023;11:1145550.
66. Zhou S-w, Wang J, Chen S-y, Ren K-f, Wang Y-x, Ji J. The substrate stiffness at physiological range significantly modulates vascular cell behavior. *Colloids Surf B Biointerfaces* 2022;214:112483.
67. Conner AA, David D, Yim EKF. The Effects of Biomimetic Surface Topography on Vascular Cells: Implications for Vascular Conduits. *Adv Healthc Mater* 2024;13:2400335.
68. Hao H, Xue Y, Wu Y, Wang C, Chen Y, Wang X, Zhang P, Ji J. A paradigm for high-throughput screening of cell-selective surfaces coupling orthogonal gradients and machine learning-based cell recognition. *Bioact Mater* 2023;28:1-11.
69. Zhang S, Sun J, Guo S, Wang Y, Zhang Y, Lei J, Liu X, Chen H. Balancing functions of antifouling, nitric oxide release and vascular cell selectivity for enhanced endothelialization of assembled multilayers. *Regen Biomater* 2024;11:rbae096.

70. Zuo X, Zhang H, Zhou T, Duan Y, Shou H, Yu S, Gao C. Spheroids of Endothelial Cells and Vascular Smooth Muscle Cells Promote Cell Migration in Hyaluronic Acid and Fibrinogen Composite Hydrogels. *Research (Wash D C)* 2020;2020:8970480.
71. Liu M, Samant S, Vasa CH, Pedrigi RM, Oguz UM, Ryu S, Wei T, Anderson DR, Agrawal DK, Chatzizisis YS. Co-culture models of endothelial cells, macrophages, and vascular smooth muscle cells for the study of the natural history of atherosclerosis. *PLoS One* 2023;18:e0280385.
72. Yang B, Li Z, Yang Z, Zhao P, Lin S, Wu J, Liu W, Yang X, Xie X, Zong Z, Lyu Y, Yang Z, Li G, Ngai T, Zhang K, Bian L. Recapitulating hypoxic metabolism in cartilaginous organoids via adaptive cell-matrix interactions enhances histone lactylation and cartilage regeneration. *Nat Commun* 2025;16:2711.
