# Supplementary material for: Migration of co-cultured endothelial and smooth muscle cells on material surfaces
Source: Regen Biomater. 2026 May 15;13:rbag087. doi: 10.1093/rb/rbag087 (PMC13229579; doi:10.1093/rb/rbag087)
Supplement: rbag087_Supplementary_Data [file rbag087_supplementary_data.zip › Supporting information_RB_Revised Ver3.pdf]

Supporting Information

Migration of co-cultured endothelial and smooth muscle cells on material surfaces

*Runjia Shen, Yanshuang Zhang, Ziyue Zhang, Qiong Liu, Jiandong Ding**

State Key Laboratory of Molecular Engineering of Polymers, Department of Macromolecular Science, Fudan University, Shanghai 200438, China

* Corresponding author. Email: jdding1@fudan.edu.cn (JD Ding)

This file includes:

Figures S1 to S7

There are 2 supplementary videos in separate MP4 files as follows:

Video S1. Collective migration of two cell types in different culture conditions recorded by a charge-coupled device equipped with a fluorescence microscope.

Video S2. Random migration of two cell types in different culture conditions recorded by a charge-coupled device equipped with a fluorescence microscope.

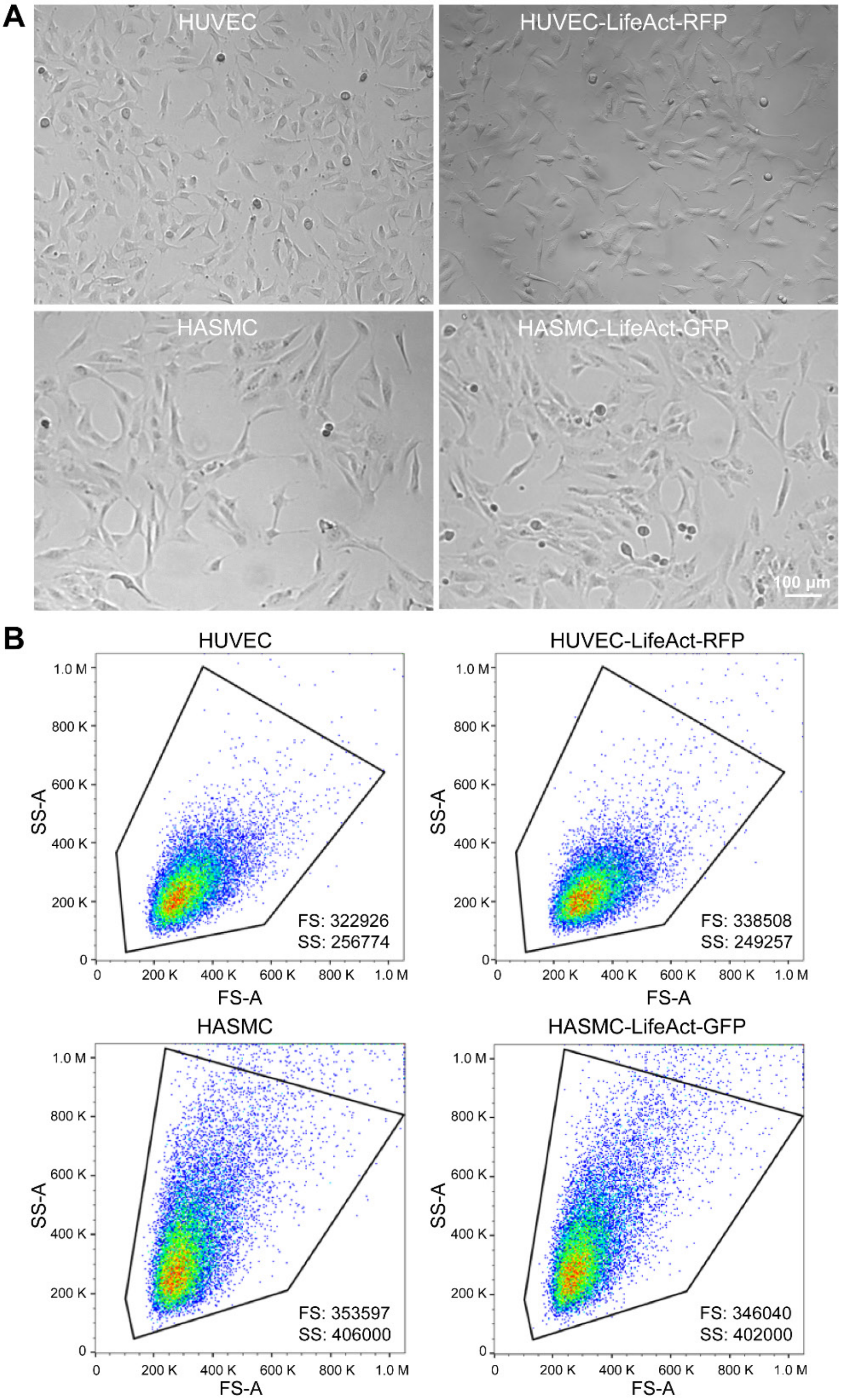

Figure S1. Morphological and physical characterization of human umbilical vein endothelial cells (EC) and human smooth muscle cells (SMC) before and after lentiviral transduction. (**A**) Representative bright-field images of ECs and SMCs before and after lentiviral transduction with LifeAct-RFP or LifeAct-GFP, respectively. Cell morphology was examined to assess potential effects of transduction. No appreciable morphological differences were observed between transduced and non-transduced cells. Scale bar: 100 μm. (**B**) Flow cytometry analysis of forward scatter (FS-A) and side scatter (SS-A) for ECs, EC–LifeAct-RFP, SMCs, and SMC–LifeAct-GFP. Comparable FS and SS distributions across groups indicate that lentiviral transduction did not significantly affect cell size or granularity.

A

Loci	STR profiling information<br>Submitted cell line:<br>**HUVEC**			STR profiling information<br>Submitted cell line:<br>**HUVEC_LifeAct_RFP**		
	Allele1	Allele2	Allele3	Allele1	Allele2	Allele3
D5S818	11	11		11	11	
D13S317	11	11		11	11	
D7S820	8	9	10	8	9	10
D16S539	11	12		11	12	
VWA	14	17		14	17	
TH01	6	8	9.3	6	8	9.3
AMEL	X	X		X	X	
TPOX	8	9		8	9	
CSF1PO	10	11	12	10	11	12
D12S391	15	18		15	18	
FGA	22	23		22	23	
D2S1338	22	24		22	24	
D21S11	28	29	32	28	29	32
D18S51	13	15	17	13	15	17
D8S1179	13	13		13	13	
D3S1358	15	16		15	16	
D6S1043	11	12	22	11	12	22
PENTAE	7	11	12	7	11	12
D19S433	13	14		13	14	
PENTAD	9	11		9	11	
D1S1656	12	17		12	17	

B

Loci	STR profiling information<br>Submitted cell line:<br>**HASMC**			STR profiling information<br>Submitted cell line:<br>**HASMC_LifeAct_GFP**		
	Allele1	Allele2	Allele3	Allele1	Allele2	Allele3
D5S818	11			11		
D13S317	9	12		9	12	
D7S820	8	11		8	11	
D16S539	9	13		9	13	
VWA	16	17		16	17	
TH01	7			7		
AMEL	X	Y		X	Y	
TPOX	8	11		8	11	
CSF1PO	12			12		
D12S391	19	21		19	21	
FGA	24			24		
D2S1338	20	23		20	23	
D21S11	28	29	30	28	29	30
D18S51	14	16		14	16	
D8S1179	14			14		
D3S1358	15			15		
D6S1043	18	20.3		18	20.3	
PENTAE	12	21		12	21	
D19S433	14	15.2		14	15.2	
PENTAD	10	13		10	13	
D1S1656						

Figure S2. Short tandem repeat (STR) profiling confirms the genetic identity of cell lines before and after lentiviral transduction. STR analysis of ECs and EC–LifeAct-RFP (**A**), as well as SMCs and SMC–LifeAct-GFP (**B**), demonstrates consistent STR profiles before and after transduction, confirming the genetic identity of the cell lines and indicating that lentiviral transduction did not alter the genomic background of the cells.

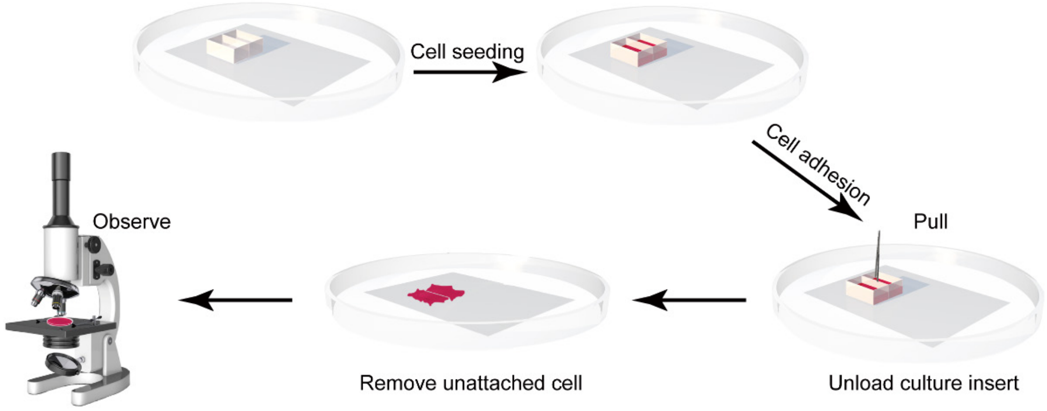

Figure S3. Schematic illustration of the experimental workflow for the collective cell migration assay on nanopatterns. A culture insert was first placed onto the biomaterial surfaces to define a confined seeding area, followed by cell seeding into the insert at a volume of 70 μL per well. After 8 h of cell adhesion, the culture insert was carefully removed to generate a well-defined cell boundary. Cell nuclei were stained with Hoechst 33342 for 5 min, after which unattached cells were removed by gentle washing. Collective cell migration from the initially confined region was then observed using a fluorescence microscope equipped with a live-cell imaging workstation.

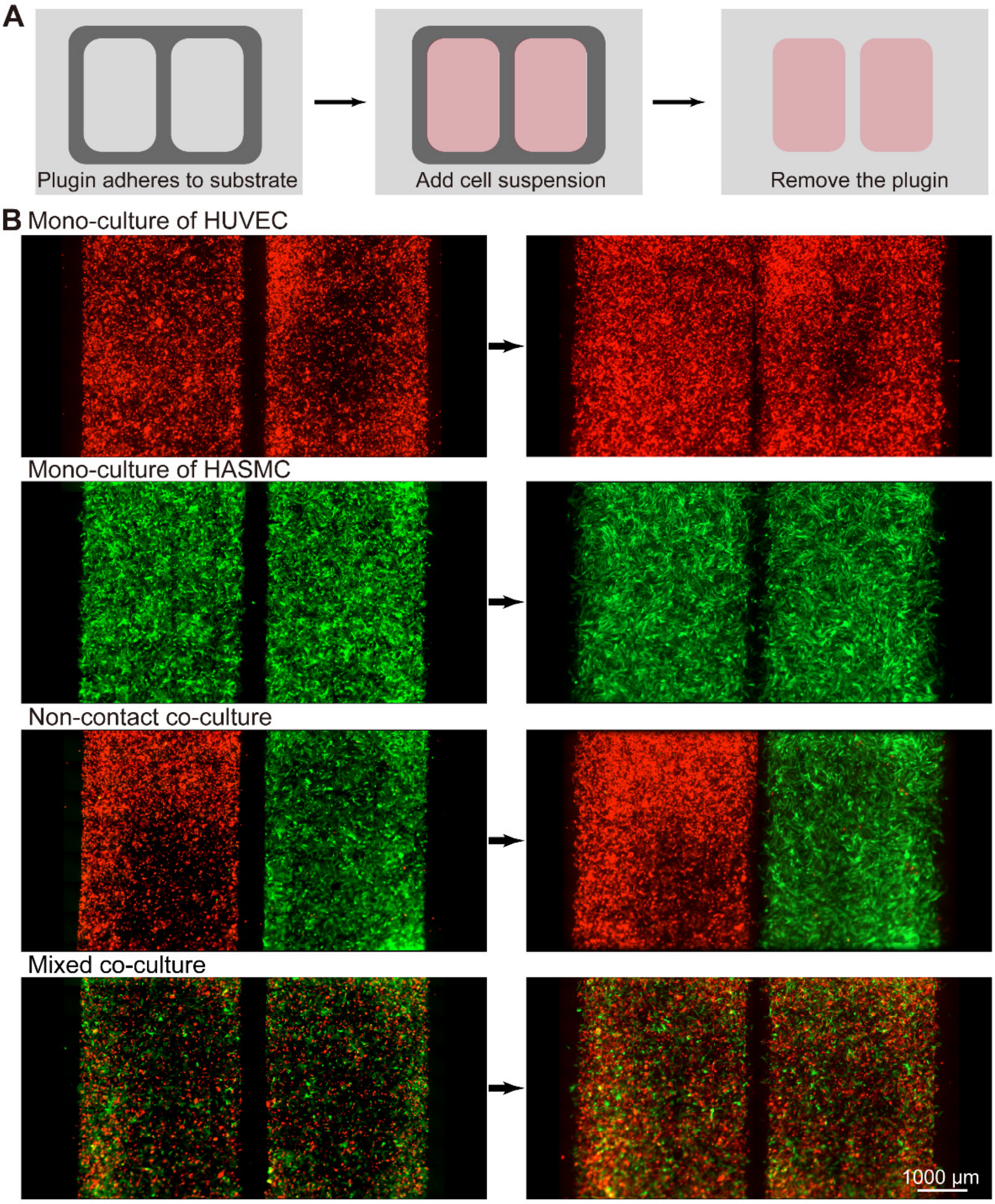

Figure S4. Stitched fluorescence micrograph of a scratch assay in a global field of view. (**A**) Schematic illustration of the experimental workflow using a removable plugin: (i) the insert is first adhered to the substrate; (ii) cell suspensions are seeded into the designated compartments; and (iii) the insert is removed to initiate a gap for the evaluation of cell migration. (**B**) Stitched fluorescence micrograph showing a large field of view of the migration assay after 12 h, illustrating collective cell migration under four conditions: EC monoculture (red fluorescence), SMC monoculture (green fluorescence), non-contact co-culture (left: EC; right: SMC), and mixed co-culture. Scale bar: 1000 μm.

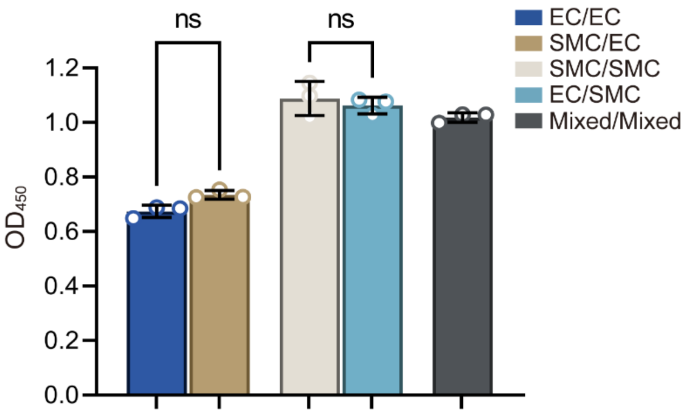

Figure S5. Quantification of cell viability measured by CCK-8 assay (OD_{450}) under different culture conditions. Five upper/lower chamber combinations were tested: EC/EC, SMC/EC, SMC/SMC, EC/SMC, and EC+SMC/EC+SMC. Cell types are indicated as upper chamber/lower chamber. The total seeding density was kept constant across all groups. Data are presented as mean ± s.d. (n = 3). No substantial differences in viability were observed among the different culture configurations, indicating that the co-culture setups did not intrinsically alter overall cell survival or metabolic activity.

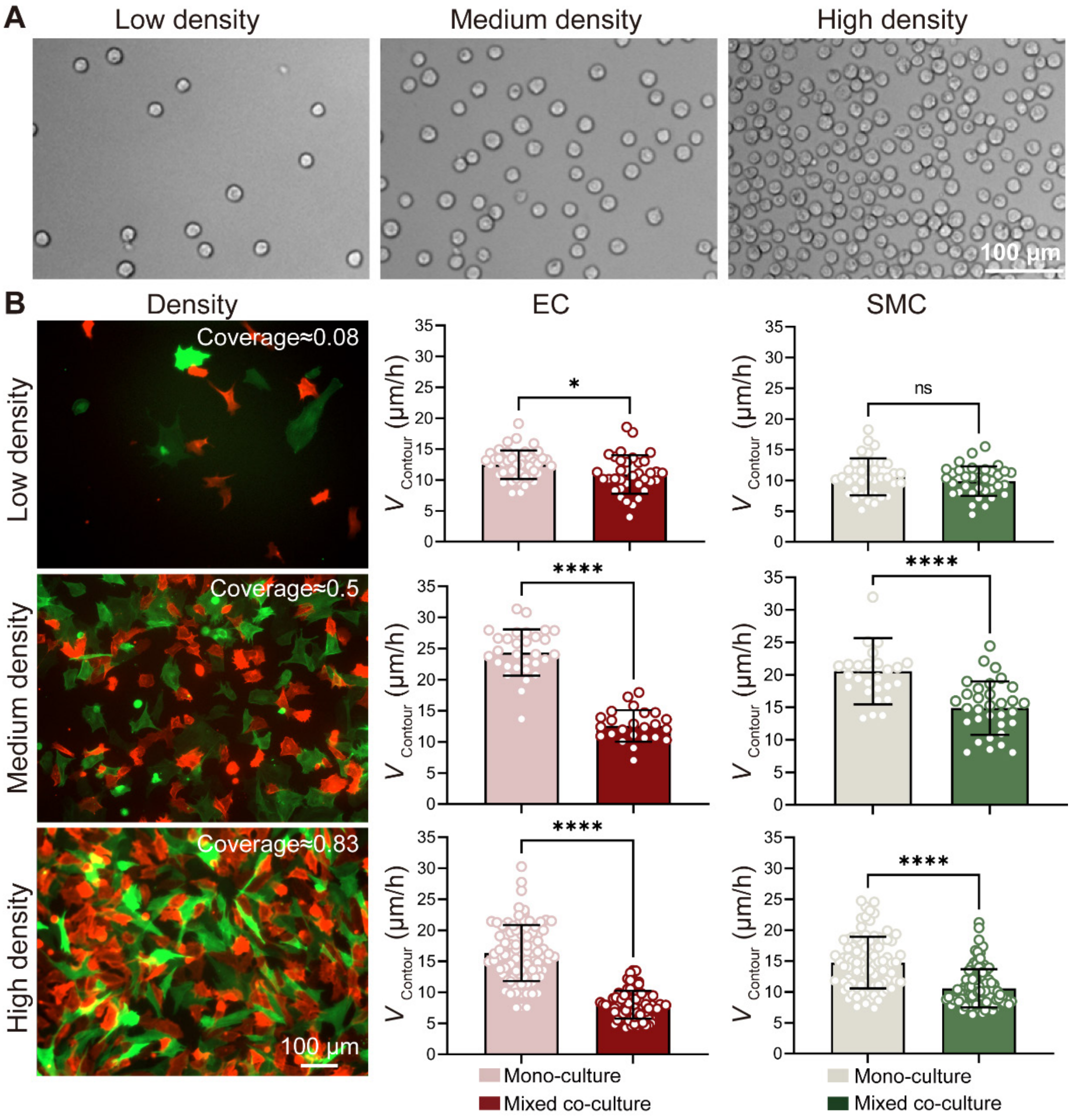

Figure S6. Co-culture suppresses migration of ECs and SMCs across all seeding densities. (**A**) Representative phase-contrast micrographs showing cell distributions at three representative seeding densities — low (5 × 10^4 cells/mL), medium (1 × 10^5 cells/mL), and high (3 × 10^5 cells/mL). Scale bar: 100 μm. (**B**) Fluorescence images and quantitative analysis of random migration speed (V_{contour}) of ECs (red) and SMCs (green) in mono-culture and direct co-culture. Across all density conditions, co-culture suppresses the random migration of both cell types compared to mono-culture. Statistical significance is indicated.

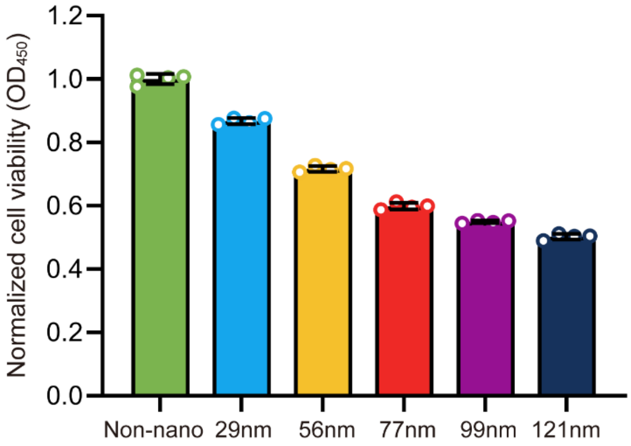

Figure S7. Relative cell viability of ECs cultured on substrates with different ligand nanospacings, quantified by CCK-8 assay. The values of optical density at 450 nm, OD_{450} were normalized to the non-nano control (set as 1). Data are presented as mean ± s.d. ($n = 4$). The overall p value among all groups was 1.03×10^{-22} (one-way ANOVA), significantly less than 0.05.
